# A Novel Feature-Map Based ICA Model for Identifying the Individual, Intra/Inter-Group Brain Networks across Multiple fMRI Datasets

**DOI:** 10.3389/fnins.2017.00510

**Published:** 2017-09-08

**Authors:** Nizhuan Wang, Chunqi Chang, Weiming Zeng, Yuhu Shi, Hongjie Yan

**Affiliations:** ^1^Neuroimaging Lab, School of Biomedical Engineering, Health Science Center, Shenzhen University Shenzhen, China; ^2^Guangdong Key Laboratory of Biomedical Information Detection and Ultrasound Imaging Shenzhen, China; ^3^Center for Neuroimaging, Shenzhen Institute of Neuroscience Shenzhen, China; ^4^Lab of Digital Image and Intelligent Computation, Shanghai Maritime University Shanghai, China; ^5^Department of Neurology, Affiliated Lianyungang Hospital of Xuzhou Medical University Lianyungang, China

**Keywords:** fMRI, feature maps, big neuroimaging data, ICA, subject-specific analysis, intragroup analysis, intergroup analysis

## Abstract

Independent component analysis (ICA) has been widely used in functional magnetic resonance imaging (fMRI) data analysis to evaluate functional connectivity of the brain; however, there are still some limitations on ICA simultaneously handling neuroimaging datasets with diverse acquisition parameters, e.g., different repetition time, different scanner, etc. Therefore, it is difficult for the traditional ICA framework to effectively handle ever-increasingly big neuroimaging datasets. In this research, a novel feature-map based ICA framework (FMICA) was proposed to address the aforementioned deficiencies, which aimed at exploring brain functional networks (BFNs) at different scales, e.g., the first level (individual subject level), second level (intragroup level of subjects within a certain dataset) and third level (intergroup level of subjects across different datasets), based only on the feature maps extracted from the fMRI datasets. The FMICA was presented as a hierarchical framework, which effectively made ICA and constrained ICA as a whole to identify the BFNs from the feature maps. The simulated and real experimental results demonstrated that FMICA had the excellent ability to identify the intergroup BFNs and to characterize subject-specific and group-specific difference of BFNs from the independent component feature maps, which sharply reduced the size of fMRI datasets. Compared with traditional ICAs, FMICA as a more generalized framework could efficiently and simultaneously identify the variant BFNs at the subject-specific, intragroup, intragroup-specific and intergroup levels, implying that FMICA was able to handle big neuroimaging datasets in neuroscience research.

## Introduction

Blood oxygen level dependent (BOLD) functional magnetic resonance imaging (fMRI) has been used as an effective neuroimaging tool to study functional connectivity, which can reveal the neural correlates of cognitive processes, among multiple cortical brain regions (Biswal et al., 1995, 1997; Kawashima et al., 2000; Greicius et al., 2003; Yang et al., 2014; Shi et al., 2015b). A recent research interest in the literature is to study functional connectivity at multiple levels using fMRI technique (Yeo et al., 2011), and for this purpose a variety of well-known methods have been utilized, e.g., general linear model (GLM; Friston et al., 1994; Bagarinao et al., 2003), clustering methods (Golay et al., 1998; Fadili et al., 2000; Cordes et al., 2002; Zhang et al., 2011; Ren et al., 2014; Tang et al., 2015), principal/independent component analysis (PCA/ICA; McKeown et al., 1998; Biswal and Ulmer, 1999; Baumgartner et al., 2000; Kiviniemi et al., 2000; Beckmann and Smith, 2004), sparse dictionary learning (Georgiev et al., 2007; Lv et al., 2015a,b; Wang et al., 2016a), etc. As a representative of the model-based methods, GLM requires a prior knowledge of design matrix. Therefore, GLM is not able to detect intrinsic brain functional networks (BFNs) at the resting state where no design matrix is available. On the contrary, since no prior knowledge on the spatial or temporal pattern prior of the BFNs is required, the data-driven methods are more widely used in functional connectivity study. Examples of such data-driven methods include spatial ICA (McKeown et al., 1998) and temporal ICA (Biswal and Ulmer, 1999), assuming the spatial and temporal independence, respectively, while probabilistic ICA (PICA) carries out a probabilistic modeling, to achieve an asymptotically unique decomposition of the fMRI data (Beckmann and Smith, 2004). Other ICA methods for fMRI data analysis include an approach making use of spatial regularity of sources (Valente et al., 2009), and the models combining the sparsity and the mutual independence of components (Calhoun et al., 2013; Wang et al., 2013, 2015), to improve the accuracy of the estimated brain sources.

In order to investigate the commonality of the functional connectivity inferred by ICAs across a group of subjects, roughly five group analysis methods have been developed by many researchers (Calhoun and Adali, 2012). The first group analysis method performs ICA on the average of the fMRI data across all subjects, with the underlying assumption that all subjects have common time courses (TCs) and spatial maps (SMs; Schmithorst and Holland, 2004). The second method, temporal concatenation group ICA model (TCGICA), performs ICA on the temporal concatenation of the fMRI data for all subjects, which allows for unique TCs for each subject but assumes common group SMs (Calhoun et al., 2001). The third one was spatial concatenation group ICA model (SCGICA), which allows for unique SMs but assumes common TCs (Svensén et al., 2002). However, for most resting-state fMRI functional connectivity studies, SCGICA does not perform so well as TCGICA (Schmithorst and Holland, 2004), possibly because the assumption of the unique time course across subjects, is more appropriate than the common-SM assumption. The fourth group ICA method called tensor-ICA concatenates the multi-subject fMRI data along a separate third dimension, and estimates a single spatial, temporal, and subject-specific mode for each component to attempt to capture a multidimensional structure of the data (Beckmann and Smith, 2005), with the assumption of both temporal and spatial consistency across the subjects. The fifth group analysis approach, makes a *post-hoc* analysis of the single-subject ICAs, to combine the components into groups by spatial correlation (Schöpf et al., 2010; Wang et al., 2012), self-organized clustering (Esposito et al., 2005), or retrospective matching of the components (Langers, 2010). Additionally, by incorporating the intragroup sources as a priori of ICA model, called ICA-R (ICA with references; Lu and Rajapakse, 2006; Shi et al., 2015a), it is expected to obtain the more accurate subject-specific brain sources. For example, a novel group information guided ICA model (GIG-ICA) with the spatial reference of the intragroup sources generated by TCGICA (Calhoun et al., 2001) was able to extract more accurate subject-specific brain sources than the traditional ICA (Du and Fan, 2013).

Though the ICA or ICA-based models have been widely used to analyze the fMRI data, the aforementioned methods have the many kinds of deficiencies. For example, the multi-step PCA operations in TCGICA, SCGICA and GIG-ICA for data reduction, possibly eliminate the subtle signals (Cordes and Nandy, 2004), which likely is not quite proper for handling the big neuroimaging data; since tensor ICA assumes common TCs among subjects, it is inappropriate for when they are different, such as in a resting-state study or when events are randomized between subjects; the single-subject ICAs also have the disadvantage that since the data are noisy, the components might not be necessarily unmixed in the same way for all subjects. Moreover, to our knowledge, these methods are just applied in BFNs identification at individual or/and intragroup levels, and there is a need to further investigate BFNs identification at intragroup-specific and intergroup levels, especially across the multiple fMRI datasets with different acquisition parameters such as variant time of repetition (TR) and different kinds of scanners. In this study, a generalized feature-map based ICA model (FMICA) is proposed to address the aforementioned deficiencies, which can be used to analyze the big fMRI datasets at individual, intragroup and intergroup levels.

The remainder of this paper is organized as follows. Theory and methods of FMICA are firstly presented in the next section, and followed by the description of the experimental designs and a subsequent section on BFN identification ability validation using both the simulation data and real fMRI datasets of task and rest at the subject-specific, intragroup and intergroup levels. Results and discussions are then presented, followed by final conclusions related to the advantages and limitations of FMICA.

## Theory and methods

In this section, the related theory of ICA and ICA-R is presented, followed by the detailed procedures of FMICA and some key issues in FMICA implementation.

### BFNs extraction and ICA/ICA-R

The BFNs extraction has been formulated as a source separation problem, based on the functional integration property of the brain (McKeown et al., 1998; Du and Fan, 2013; Shi et al., 2015a). This source separation problem can usually be divided into blind source separation (BSS) and semi-blind source separation (SBSS), depending on whether the prior is given or not. With the respect to ICA model, as a representative of BSS, it is assumed that the observed fMRI mixtures (denoted as *X*) are linearly mixed by a set of non-Gaussian sources, namely BFNs (denoted as *S*), which can be formulated as

(1) $$\begin{array}{r} X=AS \end{array}$$

The goal of ICA is to estimate an unmixing matrix *W*, such that the estimated sources *Y* computed by the following equation are good approximation of the true sources *S*:

(2) $$\begin{array}{r} Y=WX \end{array}$$

To solve Equation (1), many ICA algorithms have been proposed, e.g., the commonly used Infomax (Bell and Sejnowski, 1995) and FastICA (Hyvärinen and Oja, 1997).

ICA-R model of SBSS incorporates the prior spatial reference (denoted as *r*), and can be modeled in a constrained ICA framework, to the following constrained optimization problem

(3) $$\begin{array}{r} \text{maximize }J\left(y\right),s.t.\;g\left(y\right)\leq 0\text{ and }h\left(y\right)=E\left(y^{2}\right)-1=0, \end{array}$$

where *J*(*y*) is the contrast function of a standard ICA algorithm, and *g*(*y*) = ε(*y, r*)−ξ, with ε(*y, r*) denotes the closeness between *y* (the estimated BFN) and the reference signal *r*, and ξ signifies a threshold parameter used to restrain the distance between *y* and *r*. To solve Equation (3), the Lagrange multiplier method can be utilized to search for the solution using Newton-like learning (Lu and Rajapakse, 2006), fixed-point learning (Lin et al., 2007), or multi-object optimization (Du and Fan, 2013).

### FMICA

**Figure 1:**
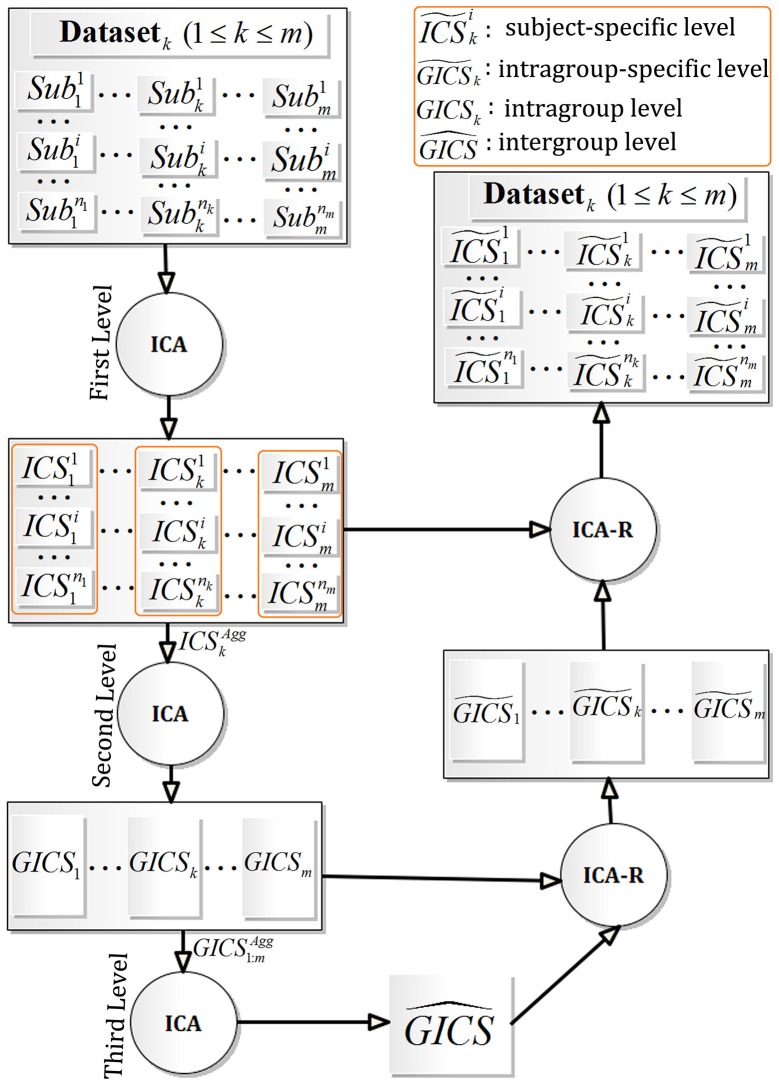
Framework of FMICA.

Supposing that there are *m* scanned fMRI datasets, i.e., Dataset_*k*_, 1 ≤ *k* ≤ *m*, the fMRI data of subject *i* in Dataset_*k*_ is denoted as $Sub_{k}^{i}$, where 1 ≤ *i* ≤ *n*_*k*_, 1 ≤ *k* ≤ *m*, and *n*_*k*_ signifying the subject number in Dataset_*k*_. As described in Figure 1, the FMICA model mainly consists of three levels of ICA decomposition and two re-estimation of group-specific and subject-specific feature maps using ICA-R: (1) the first (or single-subject) level ICA decomposition on $Sub_{k}^{i}$ to obtain the feature maps, i.e., independent components $ICS_{k}^{i}$, where 1 ≤ *i* ≤ *n*_*k*_ and 1 ≤ *k* ≤ *m*; (2) the second (or intragroup) level ICA decomposition on the aggregated feature maps, i.e., $ICS_{k}^{Agg}$ for Dataset_*k*_, to obtain the feature maps at intragroup level, i.e., *GICS*_*k*_ for Dataset_*k*_, 1 ≤ *k* ≤ *m*; (3) the third (or intergroup) level ICA decomposition on the aggregated feature maps across the datasets (Dataset_*k*_, 1 ≤ *k* ≤ *m*), i.e., $GICS_{1:m}^{Agg}$, to extract the intergroup feature maps across the different datasets, i.e., $\hat{GICS}$; (4) the ICA-R algorithm first runs on the *GICS*_*k*_ regrading Dataset_*k*_ (1 ≤ *k* ≤ *m*) to extract the correspondingly intragroup-specific feature maps, i.e., ${\tilde{GICS}}_{k}$, then on the $ICS_{k}^{i}$ regarding $Sub_{k}^{i}$ (1 ≤ *i* ≤ *n*_*k*_, 1 ≤ *k* ≤ *m*) to obtain the correspondingly subject-specific featured maps (denoted as ${\tilde{ICS}}_{k}^{i}$).

Specifically, in order to make the last procedure in FMICA more apparent, the corresponding details are described in the following. On the one hand, for extracting the intragroup-specific feature maps, i.e., ${\tilde{GICS}}_{k}$, 1 ≤ *k* ≤ *m*, the ICA-R algorithm (Du and Fan, 2013) is implemented on each *GICS*_*k*_, where the correspondingly intergroup feature maps $\hat{GICS}$ are used as the spatial references. On the other hand, in order to extract the subject-specific feature maps (${\tilde{ICS}}_{k}^{i}$) corresponding to the subject data $Sub_{k}^{i}$, 1 ≤ *i* ≤ *n*_*k*_, and 1 ≤ *k* ≤ *m*, the similar ICA-R procedure with spatial reference is implemented on each $ICS_{k}^{i}$. It is noteworthy that the spatial references may have two choices: the intergroup feature maps $\hat{GICS}$ or the corresponding intragroup-specifc feature maps ${\tilde{GICS}}_{k}$. Repeating the above procedure for Dataset_*k*_ (1 ≤ *k* ≤ *m*), all the corresponding intragroup-specific ${\tilde{GICS}}_{k}$ and subject-specific feature maps ${\tilde{ICS}}_{k}^{i}$ (1 ≤ *i* ≤ *n*_*k*_) are retrieved. However, when the number of the involved datasets is <2, i.e., *m* = 1, the third level ICA decomposition procedure is not implemented, and both the intergroup and intragroup-specific feature maps are not generated. Facing this situation, the subject-specific feature maps (${\tilde{ICS}}_{1}^{i}$) are also obtained by ICA-R, where the intragoup feature maps (*GICS*_1_) determined by the second level ICA decomposition procedure are used as the required spatial references.

Further, the corresponding statistical parametrical maps (SPMs) for ${\tilde{ICS}}_{k}^{i}$, ${\tilde{GICS}}_{k}$, $\hat{GICS}$, and *GICS*_*k*_ (1 ≤ *i* ≤ *n*_*k*_ and 1 ≤ *k* ≤ *m*) are obtained by the z-score transformation, where the BFNs are generated by threshing the corresponding SPMs with cluster size controlling.

Finally, in FMICA, it is worthy of noting that the intragroup or intergroup BFNs are identified by the second or third level ICA decomposition procedure, while the intragroup-specific or subject-specific BFNs are both identified by ICA-R procedure using the ones at the intragroup or intergroup level as references. It is expected that the BFNs at the intergroup, intragroup-specific and subject-specific levels, have some degree of similarity to each other in spatial distribution, but orderly capture the commonness across different groups, the specific activation parts of the spatial distribution within a certain group and within a single subject data. In a word, for a given intergroup BFN, the corresponding intragroup-specific or subject-specific one belongs to the same kind of BFN, but captures the group-specific or subject-specific difference in spatial distribution of BFN.

Based on the above description, FMICA is a quite generalized framework using feature maps, which is effective to capture the common BFNs (i.e., $\hat{GICS}$, *GICS*_*k*_), subject-specific ones (i.e., ${\tilde{ICS}}_{k}^{i}$) and intragroup-specific ones (i.e., ${\tilde{GICS}}_{k}$). This implies that FMICA can be used to not only explore the subject-specific differences within a group, but also to reveal the intragroup-specific differences across the multiple datasets.

### Some key points in FMICA implementation

With respect to the FMICA implementation, the number of independent components (IC) in ICA decomposition at different levels should be first addressed. For the first level ICA decomposition procedure (depicted in Figure 1), the Laplace approximation (Minka, 2000) previously used in probabilistic ICA for fMRI data analysis (Beckmann and Smith, 2004) is used to estimate the components number for each single-subject fMRI data; for the second level ICA decomposition, the mean order corresponding to all subjects within the same dataset is used, while the average order is usually used as the number of intragroup level components in TCGICA (Calhoun et al., 2001; Li et al., 2007) implemented in the GIFT software (http://mialab.mrn.org/software/gift/index.html); for the third level ICA decomposition, the average number of components in *GICS*_*k*_, 1 ≤ *k* ≤ *m* is used due to the situation of the different session scans of the same subjects under the same condition (for example, Experiment 2 of Section Experimental Designs); otherwise, the stability measure retrieved by ICASSO (Himberg et al., 2004) is used to determine the optimal components number, where ICASSO runs from the minimum number (i.e., min{*x*|*x* = #(*GICS*_*k*_), 1 ≤ *k* ≤ *m*}) to the maximum one (i.e., max{*x*|*x* = #(*GICS*_*k*_), 1 ≤ *k* ≤ *m*}) to obtain the stability values under different order number. Specifically, #() is an operation of obtaining the components number of the intragroup feature maps *GICS*_*k*_, 1 ≤ *k* ≤ *m* (for example, Experiment 3 of Section Experimental Designs). Moreover, in this research, FMICA takes advantage of the FastICA (Hyvärinen and Oja, 1997) and GIG-ICA (Du and Fan, 2013), to perform the ICA decomposition and ICA-R decomposition, respectively. Additionally, since the performance of ICA-R depends on the accuracy of the spatial references (Du and Fan, 2013; Wang et al., 2014; Shi et al., 2015a), slightly thresholded feature maps which are more similar to the real activated BFNs are used as spatial references, where the corresponding z threshold value is set to 1.0 empirically. Finally, the z-threshold value and the cluster size threshold are set to 2.0 and 10 voxels, respectively, to obtain the ultimate BFNs.

## Experimental tests

In this section, the efficacy of the proposed FMICA model, was validated on the simulation data, task-related fMRI data and resting-state fMRI data. The details of the designed experiments were presented as follows.

### Experimental datasets

#### Simulation dataset

The SimTB toolbox (http://mialab.mrn.org/software; Allen et al., 2012; Erhardt et al., 2012) was used to generate simulation dataset including 20 subjects. Each subject data was with V = 148 × 148 voxels, 12 spatial sources and 120 time points at TR = 2 s (s). The baseline intensity was set to 800, and the baseline map was shown in Figure S1A. Each source, depicted in Figure S1B, represented a spatial pattern that underwent certain activation over time. Two sources (10 and 12) shared task-related modulation in addition to having unique fluctuations. For source 10, the strength of task-modulation (expressed as the ratio between task event amplitude and unique event amplitude) was set to 4, while task-relatedness was smaller for source 12, set to 2. Task-modulation was introduced with a block design (24 s on, 24 s off, five blocks), convolved with a canonical hemodynamic response function to simulate the slow dynamics of the vascular response (Friston et al., 1995). Activation for the other 10 sources was described solely unique hemodynamic fluctuations with no task-related variation. All sources had unique events that occurred with a probability of 0.2 at each TR. For task-modulated sources (10 and 12), unique events were added with smaller amplitudes (0.2 and 0.4, respectively). For sources not of interest (no task modulation), the unique amplitude was 1. For all sources, the percent signal change was centered at 3 with a standard deviation of 0.25. Additive noise was included to reach a specified contrast-to-noise ratio of 1. The time courses corresponding to the simulated 12 sources were depicted in Figure S1C. To simulate the subject-specific variations in spatial domain, modifications such as translation, rotation, expansion, and contraction, were also randomly added to each source of each subject, where the corresponding parameters were depicted in Figure S1D.

#### Visual task dataset

Six subjects (4 males and 2 females) took part in this visual task experiment, all informed about the purpose of this study and all the participants included in this study provided written informed consent according to procedures approved by the IRB of East China Normal University (ECNU). The designed visual paradigm was a two-states (*OFF, ON*) × 3 block design with a duration of 40 s. At the “ON” state, visual stimulus was corresponding to a radial blue/yellow checkerboard, reversing at 7 Hz. While at the “OFF” state, the participants were required to focus on the cross presented at the center of the screen. The BOLD fMRI data were acquired in the Shanghai Key Laboratory of Magnetic Resonance of ECNU, on a Siemens 3.0 Tesla scanner with a gradient echo EPI with 36 slices providing whole-brain coverage, TR = 2.0 s, scan resolution = 64 × 64, in-plane resolution = 3.75 × 3.75 mm; the slice thickness was 4 mm; and the slice gap was 1 mm. This dataset was also used in our previous study (Ren et al., 2014).

#### Test-retest task-related datasets for motor, language, and spatial attention

This test-retest fMRI datasets for motor, language and spatial attention functions were downloaded from the openfmri website (https://openfmri.org/dataset/; Gorgolewski et al., 2013). Three task-related fMRI time series (motor, covert verb generation, and landmark tasks) were selected to validate our proposed FMICA model. Ten healthy subjects (median age 52.5 years, min = 50, max = 58) included four males and six females, of which three were left-handed and seven right-handed. Each subject was scanned twice, either 2 (eight subjects) or 3 (two subjects) days apart. All subjects were provided with the written informed consent and this study was approved by South East Scotland Research Ethics Committee 01. The fMRI data acquisition parameters were set as follows: GE Signa HDxt 1.5T MRI scanner, FOV = 256 × 256 mm, in-plane matrix = 64 × 64, slice thickness = 4 mm, slice number = 30, TR = 2.5 s, flip angle = 90°. The number of volumes in time series regarding the motor, covert verb generation and landmark tasks were 173, 184, and 238, respectively. For the convenience of description, the motor, covert verb generation and landmark tasks were denoted as Task1, Task2, and Task3, respectively.

#### Test-retest NYU resting-state datasets

The test-retest resting-state fMRI datasets with 25 normal participants were drawn from the Human Connectome Project (http://www.nitrc.org/projects/nyu_trt; Zuo et al., 2010). All the participants included in this study were provided with written informed consent according to procedures approved by the IRB of New York University (NYU). Also, the fMRI data were collected according to protocols approved by the institutional review boards of NYU and the NYU School of Medicine. Each participant was scanned three times at rest by a Siemens Allegra 3.0 Tesla MRI scanner and the fMRI data for each subject consisted of 197 contiguous EPI functional volumes (TR = 2 s, TE = 25 ms, flip angle = 90°, slice number = 39, matrix = 64 × 64, FOV = 192 × 192 mm^2^, acquisition voxel size = 3 × 3 × 3 mm^3^). Data of sessions 2 and 3 were collected 5–16 months (mean 11 ± 4 months) after session 1 with an interval of 45 min. A high-resolution T1-weighted magnetization prepared gradient echo sequence was also obtained for each participant (MPRAGE, TR = 2.5 ms, TE = 4.35 ms, TI = 900 ms, flip angle = 8°, slice number = 176, FOV = 256 × 256 mm^2^).

### Data preprocessing

All computations of this study were performed on a personal computer with intel(R) Core(TM) i5-3210M 2.5 GHz CPU and 4 GB RAM. The operation system platform was Windows 7. All steps for preprocessing or processing were run on the Matlab platform (Matlab 2012b, Mathworks Inc., Sherborn, MA, USA).

No preprocessing step was involved for the simulation dataset; For the other real data, the widely used DPARSF (Yan and Zang, 2010) batch processing pipeline with embedding SPM8 software (http://www.fil.ion.ucl.ac.uk/spm/) was used to perform the preprocessing operations including slice-timing, motion correction, spatial normalization to the Montreal Neurological Institute (MNI) EPI template and spatial smoothing with the full width at half maximum (FWHM) equal to 6 mm. Specifically, considering magnetization equilibrium, the first ten volumes were discarded for the test-retest NYU datasets. For the task-related datasets, no first volumes were discarded.

The z threshold of the z-scored SPMs from all the real fMRI datasets was set to 2.0, and the least active cluster size was set to 10 voxels. The BFNs were displayed by the MRIcroN software (https://www.nitrc.org/projects/mricron), and their locations were assessed by the PickAtlas toolbox (Maldjian et al., 2003, 2004).

### Experimental designs

Three kinds of experiments were designed to validate the effectiveness of FMICA in this study.

Experiment 1: the simulation dataset with only one session was used to validate effectiveness of FMICA in two aspects, i.e., the BFN identification ability at the subject-specific and intragroup levels. Specifically, the third level ICA step was not involved in this experiment. The corresponding pipeline consisted of three procedures: the first level ICA on simulation dataset for obtaining the initial FMs (ICs), the second level ICA for extracting the second level (or intragroup) FMs and the ICA-R procedure using the intragroup FMs as the references for obtaining the subject-specific BFNs, respectively.

Experiment 2: the NYU resting-state datasets with three sessions were used to validate effectiveness of FMICA in three aspects, i.e., the BFN identification ability at the subject-specific, intragroup-specific and intergroup levels. The corresponding pipeline consisted of the following steps: the first level ICA on fMRI datasets of each rest session for obtaining the initial FMs, the second level ICA for extracting the second level FMs, the third level ICA for obtaining the intergroup FMs and the ICA-R using the intergroup FMs as the references for obtaining the intragroup-specific and subject-specific FMs, respectively.

Experiment 3: the ability of capturing the group difference of intrinsic BFNs across the multiple kinds of datasets using FMICA was validated using a combination of the test-retest NYU resting-state datasets, the test-retest task-related datasets for motor, language, and spatial attention and the visual task dataset. The corresponding pipeline consisted of the following steps: the first level ICA on each aforementioned dataset for obtaining the initial FMs, the second level ICA for extracting the intragroup FMs, the third level ICA for retrieving the intergroup FMs and the ICA-R procedure using the intergroup FMs as the references for obtaining the intragroup-specific FMs.

## Results and analysis

### Results of experiment 1

**Figure 2:**
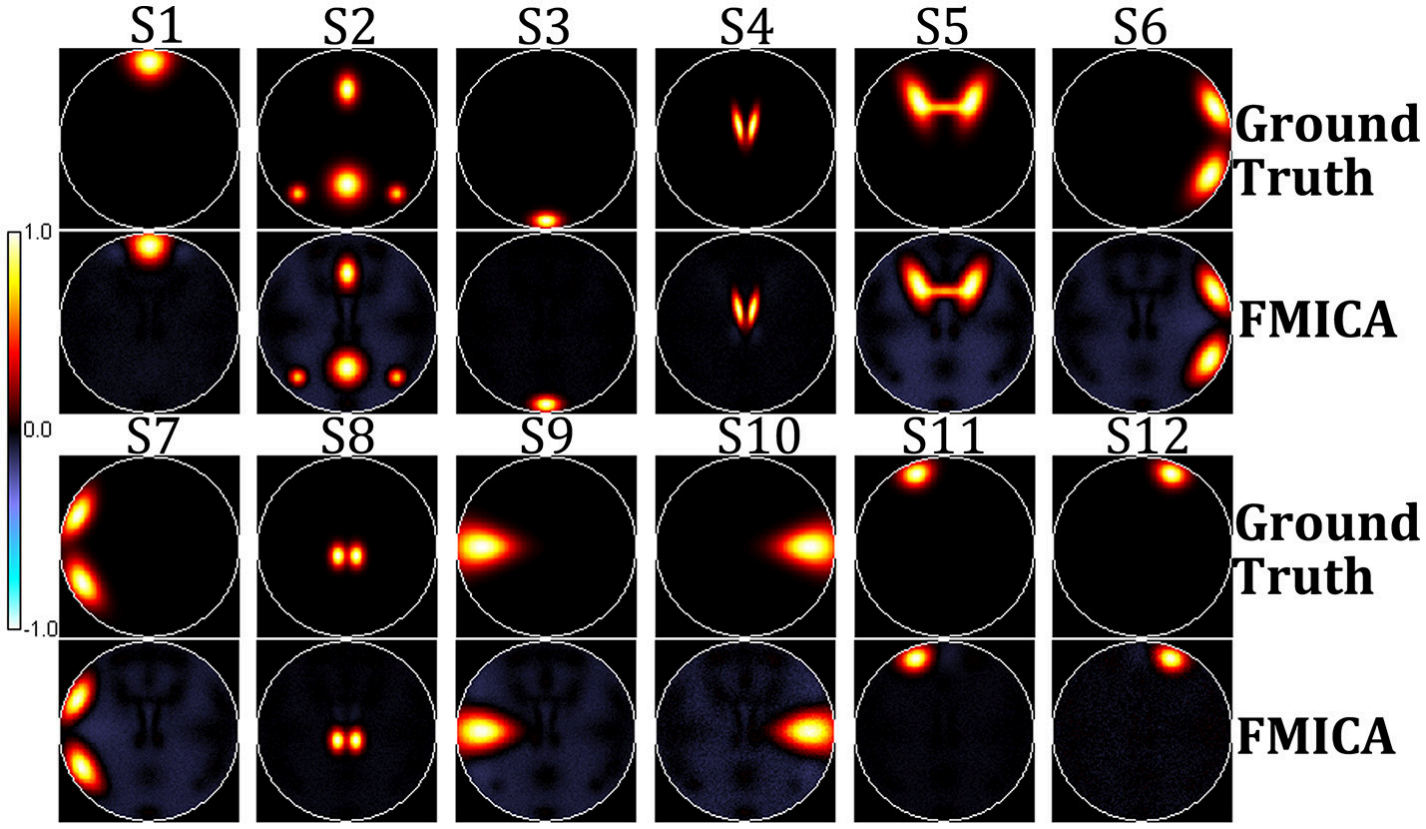
The simulated ground truth sources and the intragroup sources estimated by FMICA.

According to Experiment 1, the BFN detection ability of FMICA at the subject-specific and intragroup levels was designed to be validated on the simulation dataset. The order in both first (individual) and second (intragroup) level ICA decomposition was set to 13 (twelve designed sources and one background source). Firstly, the 12 sources determined by FMICA at intragroup level were displayed in Figure 2, which were highly approximate to the simulated ground truth sources. The Pearson correlation coefficients between the 12 estimated intragroup sources and the corresponding 12 ground truth sources were 0.9783, 0.9672, 0.989, 0.9858, 0.9634, 0.9687, 0.9687, 0.9877, 0.9717, 0.9670, 0.9868, and 0.9703, respectively, quantitatively implying the effectiveness of FMICA in the intragroup BFNs identification. Moreover, in order to investigate the intragroup BFNs estimation from the different ratios of the retained ICA components of the intragroup level to that of the individual level, FMICA was performed with a variety of such intragroup-to-individual ratios on the simulation dataset to obtain the intragroup-level BFNs, and then the mean and standard deviation (std) values of Pearson correlation coefficients between the estimated intragroup sources and the corresponding ground truth sources at each run were calculated. As shown in Table S1, from which, one could draw a conclusion that increasing the number of retained components at the individual level had no effect on performance, while greatly increasing the number of retained components at intragroup level had a certain degree of negative impact on the estimated intragroup sources, possibly due to the over-spilt effects in ICA decomposition in the simulation dataset. Table S1 also demonstrated that using the 13 components in both first-level and second-level ICA exhibited good BFNs identification performance in this simulation dataset.

**Figure 3:**
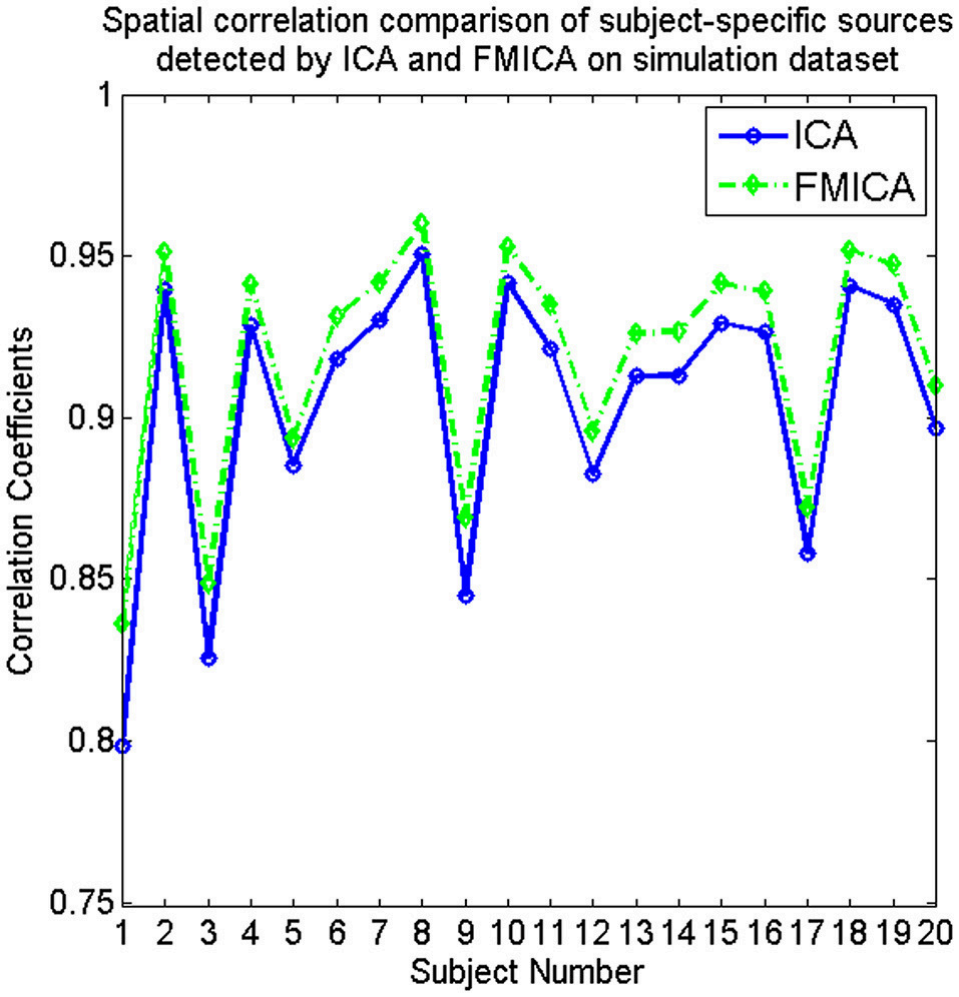
The comparative curves of spatial correlation regarding the subject-specific sources identified by ICA and FMICA, respectively, for the simulated 20 subjects on simulation dataset.

The subject-specific BFNs for each simulated subject were also estimated by FMICA, and then the correlation coefficients between these estimated subject-specific BFNs and the corresponding ground truth sources for each subject were also calculated, with the mean correlation coefficient across the 12 sources for each subject denoted as its subject-specific BFNs identification accuracy, which was compared to those of the traditional ICA model, demonstrating superior subject-specific BFNs identification ability as shown in Figure 3.

### Results of experiment 2

In this experiment, the test-retest NYU resting-state datasets with three sessions were used to validate effectiveness of the proposed FMICA model on identifying the BFNs at the intergroup, intragroup-specific, and subject-specific levels. At the subject-specific level, compared to the intrinsic BFNs from different subjects, the intrinsic BFNs originated from the different sessions of the same subject should be more correlated. Moreover, the intragroup-specific intrinsic BFNs from different sessions should be also highly correlated with each other due to the high reproducibility of the intrinsic BFNs (Shehzad et al., 2009; Zuo et al., 2010; Wang et al., 2016b).

**Figure 4:**
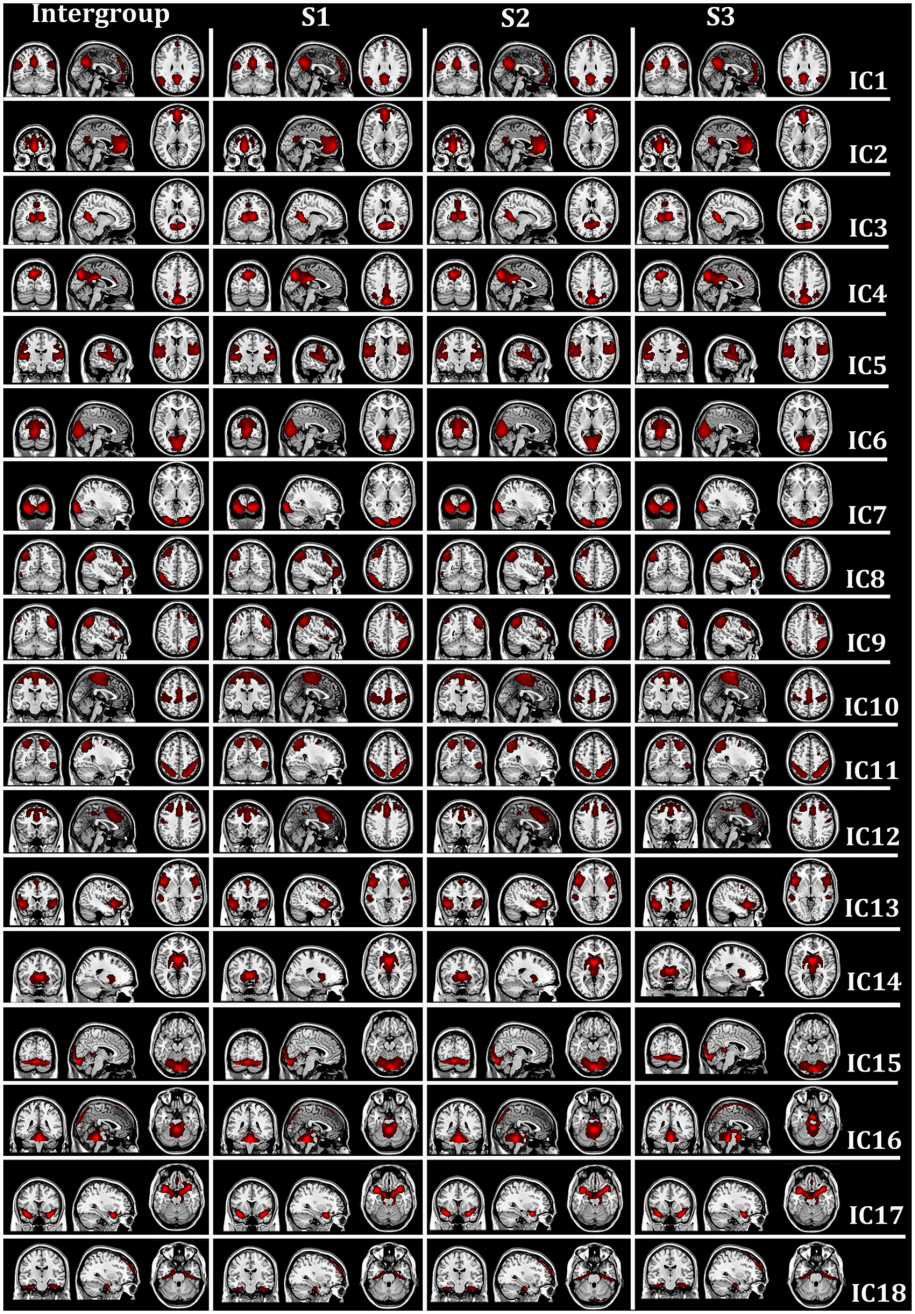
The spatial map distribution of the intrinsic BFNs at the intergroup and intragroup-specific levels on test-retest resting-state datasets: the first column depicted the intergroup intrinsic BFNs from the three rest sessions; the second, third, and fourth columns displayed the intragroup-specific intrinsic BFNs from the first (S1), second (S2), and third (S3) rest session, respectively.

Eighteen intrinsic BFNs at the intergroup and intragroup-specific levels for the resting session 1 (S1), session 2 (S2), and session 3 (S3) were selected visually by the experts from the estimated components by FMICA, as displayed in Figure 4, respectively, with the involved Talairach Daemon (TD) lobes, Brodmann areas, Automated Anatomical Labeling (AAL) atlas regions, and the representative MNI coordinates, presented in Table 1. The components IC1-IC4 were referred to the well-known default mode network (DMN; Raichle et al., 2001; Damoiseaux et al., 2006; De Luca et al., 2006), which were divided into four sub-networks (Zuo et al., 2010); IC5, IC6, IC7, and IC10 were called the auditory network, predominant visual network, lateral visual network, and sensorimotor network, respectively (Beckmann et al., 2005; Damoiseaux et al., 2006; De Luca et al., 2006; Schöpf et al., 2010; Wang et al., 2012); IC8 and IC9 were involved with the working memory function related brain regions (Wang et al., 2012, 2013; Iraji et al., 2016); IC11 and IC12 involved dorsal parietal and lateral prefrontal cortex, which were two split separate components of a dorsal pathway network (Damoiseaux et al., 2008; Schöpf et al., 2010; Wang et al., 2012, 2013); IC13 was the salience network as reported by Menon and Uddin (2010) and Uddin (2015); IC14 was the basal ganglia network, involving mainly caudate nucleus and putamen (Iraji et al., 2016); IC15 involved the cerebellum posterior lobe and a portion of calcarine area in the occipital lobe; IC16 was located at the brainstem and cerebellum, e.g., cerebellar vermis; IC17 involved mainly brodmann areas 47 and 34, e.g., the superior temporal pole; IC18 was located at a portion of the limic and frontal cortex, e.g., hippocampus, some areas of superior frontal gyrus, etc. The successful identification of the aforementioned well-known intrisic BFNs at the intergroup and intragroup-specific levels demonstrated the effectiveness of the proposed FMICA model.

**Table 1:** The location information of the 18 intrinsic BFNs from the test-retest resting-state datasets shown in Figure 4: the MNI coordinates (in mm), the involved brain lobes, Brodmann areas and AAL atlas regions for each network.

**IC number**	**Representative MNI coordinates (X, Y, Z) in mm**	**TD lobes/Brodmann areas/AAL atlas**
IC1	0, −57, 30	Parietal Lobe, Frontal Lobe/brodmann areas 7, 40 9/Frontal_Sup_Medial_L, Frontal_Sup_Medial_R, Angular_R, Precuneus_R, Precuneus_L, Angular_L
IC2	−2, 55, 7	Frontal Lobe, Limbic Lobe/brodmann areas 10, 31/Frontal_Sup_Medial_L, Precuneus_L
IC3	11, −56, 18	Limbic Lobe, Parietal Lobe/brodmann area 23/Precuneus_R, Precuneus_L
IC4	−2, −77, 41	Parietal Lobe/brodmann area 7/Precuneus_L
IC5	−58, −15, 11	Temporal Lobe/brodmann areas 42, 41/Temporal_Sup_L
IC6	3, −83, 6	Occipital Lobe/brodmann area 18/Calcarine_R
IC7	28, −94, −1	Occipital Lobe, Middle Occipital Gyrus/brodmann area 17/Occipital_Sup_L, Occipital_Sup_R, Occipital_Mid_L
IC8	−43, −56, 51	Parietal Lobe, Frontal Lobe/brodmann areas 7, 8/Frontal_Mid_L, Parietal_Inf_L
IC9	50, −53, 46	Parietal Lobe, Frontal Lobe/brodmann area 40/Frontal_Mid_R, Parietal_Inf_R
IC10	0, −19, 53	Frontal Lobe/brodmann areas 3, 4/Supp_Motor_Area_R, Postcentral_L, Precentral_R
IC11	27, −56, 53	Parietal Lobe/brodmann area 7/Parietal_Inf_L, Parietal_Inf_R
IC12	1, 19, 40	Frontal Lobe/brodmann areas 32, 9/Cingulum_Mid_R, Cingulum_Mid_L, Frontal_Mid_L, Frontal_Mid_R
IC13	−44, 20, −3	Frontal Lobe/brodmann areas 47, 6/Frontal_Inf_Orb_L, Frontal_Inf_Tri_L, Frontal_Inf_Orb_R, Supp_Motor_Area_L
IC14	−20, 7, 6	Sub-lobar/Putamen/Caudate_L, Putamen_R, Putamen_L
IC15	6, −80, −17	Occipital Lobe, Cerebellum Posterior Lobe/brodmann area 18/Calcarine_L, Vermis_6, Cerebelum_6_R, Cerebelum_6_L
IC16	2, −39, −25	Midbrain, Brainstem, Cerebellum/Vermis_1_2, Vermis_4_5, Cuneus_L
IC17	31, 13, −20	Frontal Lobe, Limbic Lobe/brodmann areas 47, 34/Insula_R, Temporal_Pole_Sup_L
IC18	32, −17, −27	Limbic Lobe, Frontal Lobe/brodmann area 10/ParaHippocampal_R, Hippocampus_L, Frontal_Mid_R, Frontal_Sup_R

**Figure 5:**
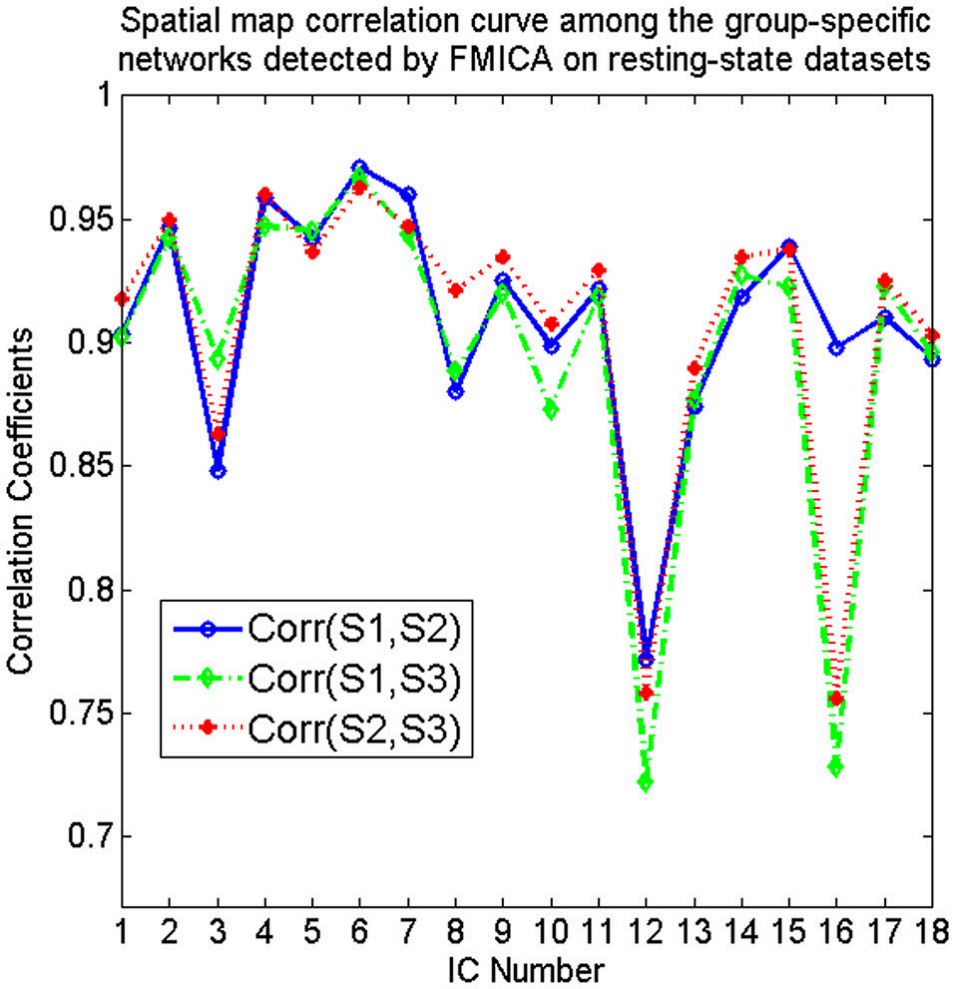
The spatial map correlation curves among the correspondingly intragroup-specific intrinsic BFNs identified by FMICA from the first (S1), second (S2), and third (S3) session of resting-state datasets, respectively.

From Figure 4, it could be observed that the intrinsic BFNs at the intragroup-specific level from each session were quite approximate to the corresponding ones at the intergroup level. Meanwhile, three pairs of mutual correlations among the intragroup-specific BFNs estimated from the three sessions, were calculated, as shown in Figure 5, from which high correlations could be clearly observed, demonstrating great reproducibility of the intrinsic BFNs across the sessions.

**Figure 6:**
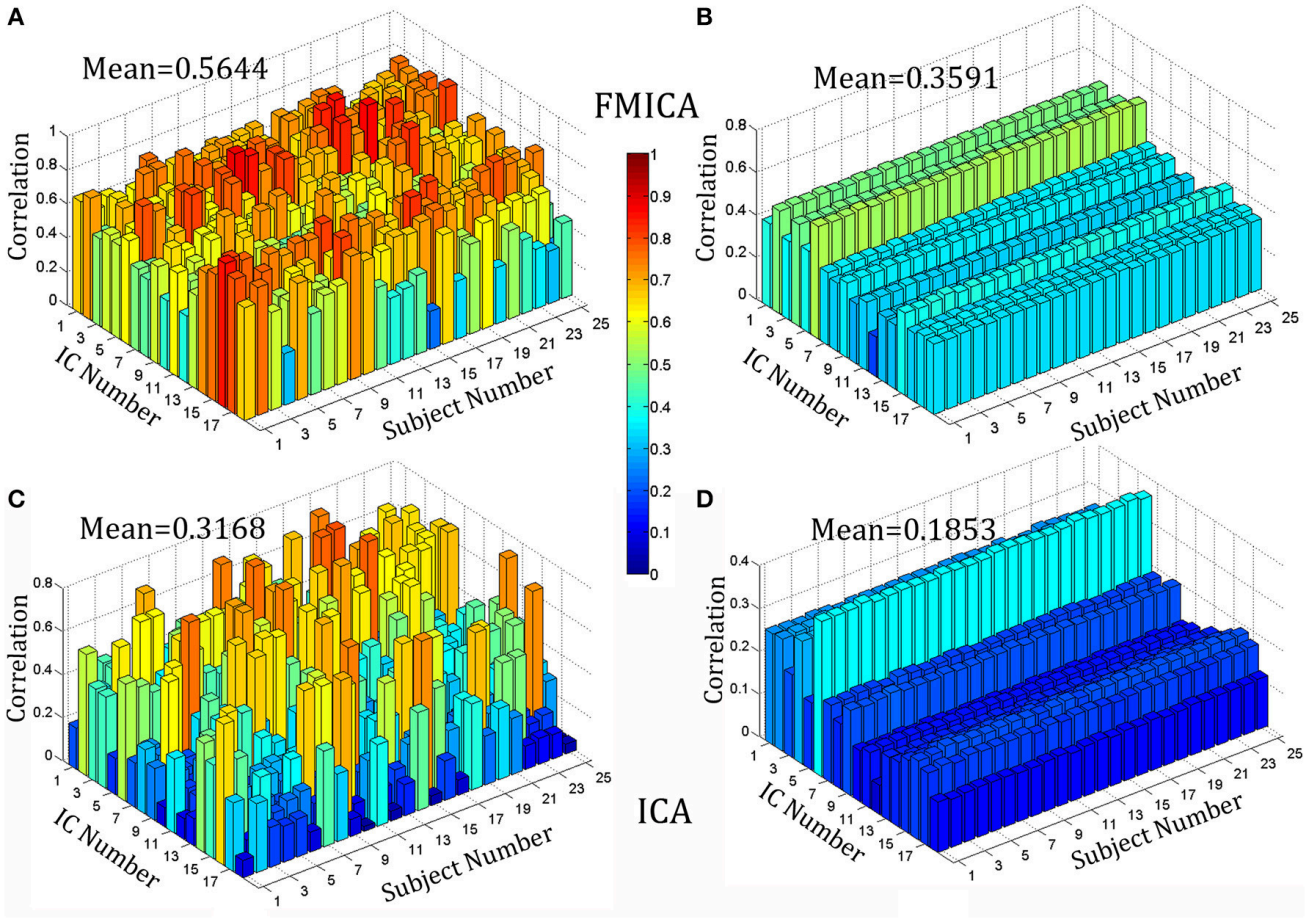
The correlation bar chart of 18 intrinsic BFNs against 25 subjects on the test-retest resting-state datasets: **(A)** denoted the mean across-sessions correlation among the intrinsic BFNs estimated by FMICA for the different sessions of the same subject; **(B)** denoted the mean across-subjects correlation among the intrinsic BFNs estimated by FMICA for the different subjects; **(C)** denoted the mean across-sessions correlation among the intrinsic BFNs estimated by FastICA for the different sessions of the same subject; **(D)** denoted the mean across-subjects correlation among the intrinsic BFNs estimated by FastICA for the different subjects.

At the subject-specific level, the BFNs identified by FMICA were compared among sessions of the same subject and among different subjects, respectively, and mean correlation coefficients between pairs of BFNs under comparison for each subject were shown in Figure 6, where Figures 6A,B were for across-sessions and across-subjects comparison, respectively. Similar to FMICA, the BFNs identified by the first level ICA were also compared among sessions of the same subject and among different subjects, respectively, and mean correlation coefficients between pairs of BFNs under comparison for each subject were also shown in Figures 6C,D for across-sessions and across-subjects comparison, respectively. It was worth noting that the 18 intrinsic BFNs at the intergroup level were used as the templates to match the best ones from the individual FMs generated by the first level ICA decomposition on each session data of each subject, aiming at overcoming the random order of the components. Moreover, based on the contrast values presented in Figures 6A,C, and the contrast ones in Figures 6B,D, two sample *T-*tests with significance level equal to 0.05 were implemented, respectively, where the mean value (0.5644, marked in Figure 6A) of all points in Figure 6A was significantly larger than the one (0.3168, marked in Figure 6C) of all points in Figure 6C with p value equal to 6.1630 × e^−80^, and the mean value (0.3591, in Figure 6B) of all points in Figure 6B was also significantly larger than the one (0.1853, marked in Figure 6D) of all points in Figure 6D with *p* value equal to 2.1511 × e^−172^. The mean values of the points in Figures 6B,D corresponding to the first level ICA were relatively small, and this was possibly due to that some BFNs could be identified at the intergroup level, but not separated at the single subject level by the traditional ICA. However, the ICA-R re-estimation procedure in FMICA could identify most of BFNs at the single subject level, demonstrating that the proposed FMICA could identify the subject-specific BFNs more effectively than the traditional ICA did.

**Figure 7:**
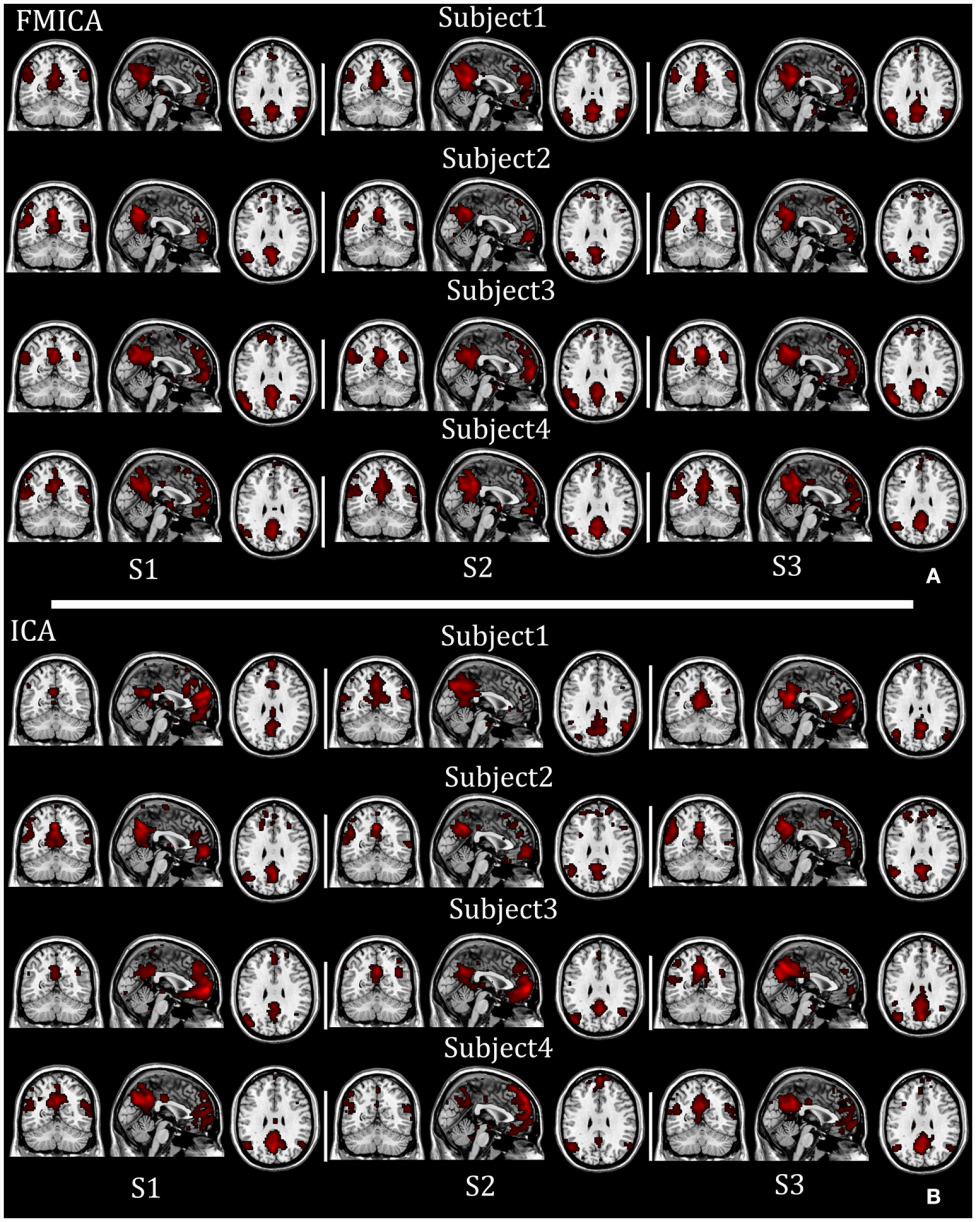
The individual DMNs identified by FMICA and FastICA on the test-retest resting-state fMRI datasets with respect to four examplars: **(A)** subject-specific DMNs identified by FMICA; **(B)** subject-specific DMNs identified by FastICA.

To intuitively compare the performance between the proposed FMICA and the traditional ICA, the spatial maps of IC1 (DMN) identified by FMICA and FastICA for all three sessions of the first four subjects (for space limitation) as shown in Figures 7A,B, respectively, were taken as an example, from which it was obvious that the DMNs identified by FMICA had higher across-sessions than across-subjects consistency and much higher both across-sessions and across-subjects consistency compared to the results of FastICA, implying that FMICA had higher subject-specific BFNs identification capability than traditional ICA.

In summary, results from the test-retest resting-state datasets demonstrated that the proposed FMICA model had high BFN identification capability at intergroup, intragroup-specific and subject-specific levels.

### Results of experiment 3

In this experiment, the intergroup and intragroup-specific analysis ability of FMICA was further validated by combining the test-retest resting-state datasets, the test-retest task-related datasets for motor, language and spatial attention (i.e., Task1, Task2, and Task3) and the visual task dataset. Namely, there were ten datasets as the input of FMICA, i.e., the resting-state datasets with three sessions, three test-retest task-related datasets (i.e., Task1, Task2, and Task3) with two sessions and the visual task dataset with one session. In this experiment, as described in Section Some Key Points in FMICA Implementation, the ICASSO method was used to determine the optimal order for the intergroup-level analysis based on the stability measure of the estimated components for each order, as shown in Figure S2, demonstrating that the estimated components had the highest mean/median stability and relatively small values of standard deviation (STD) and inter-quartile range (IQR), when the order was equal to 57. Therefore, the order was set to 57 in Experiment 3.

**Figure 8:**
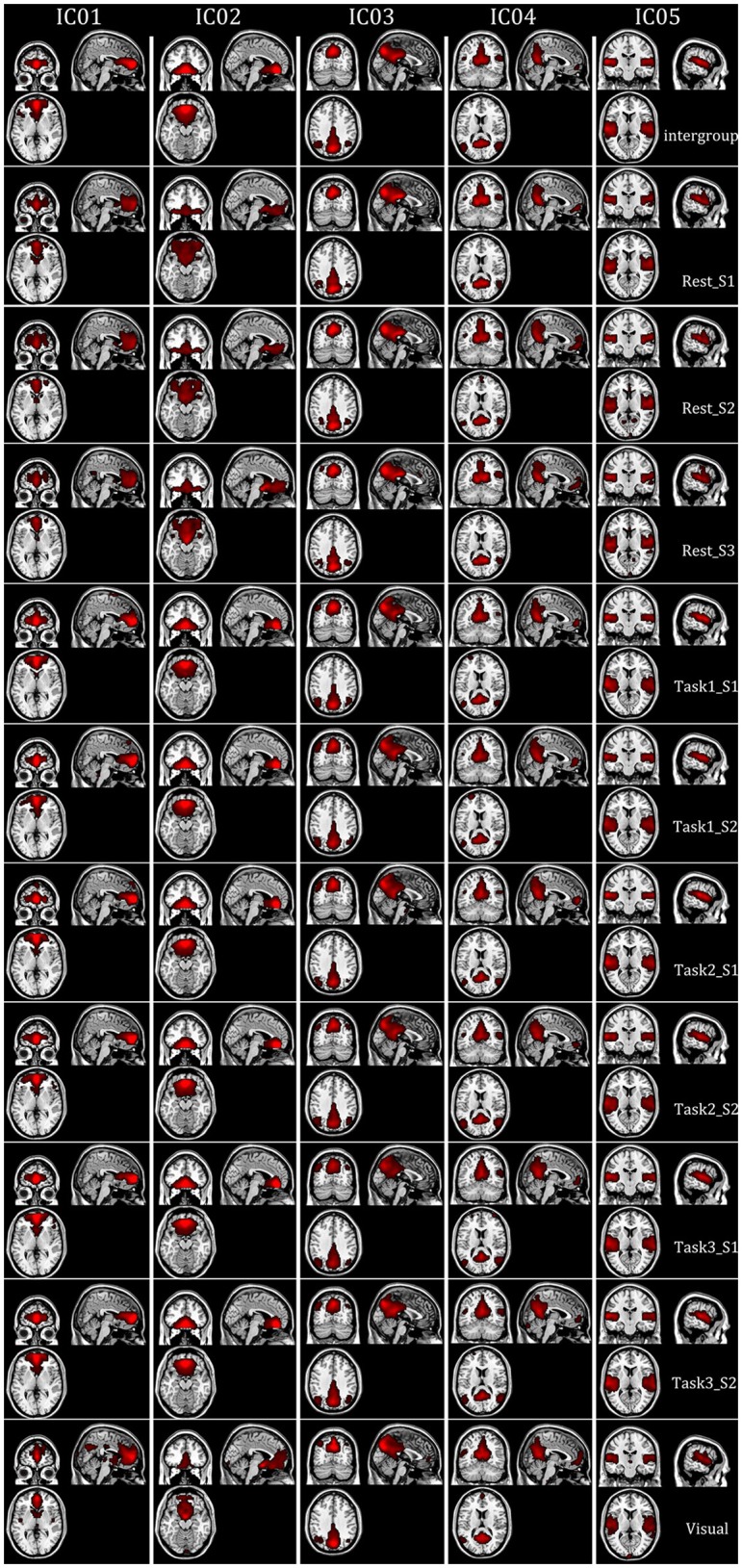
The spatial map distribution of first five BFNs at the intergroup and intragroup-specific levels in Experiment 3: each column depicted a BFN at the intergroup and intragroup-specific levels; Rest_S i denoted the ith session of test-retest resting-state datasets; Task i_S j denoted the jth session of Task i from the test-retest task-related datasets; Visual denoted the visual task dataset.

**Figure 9:**
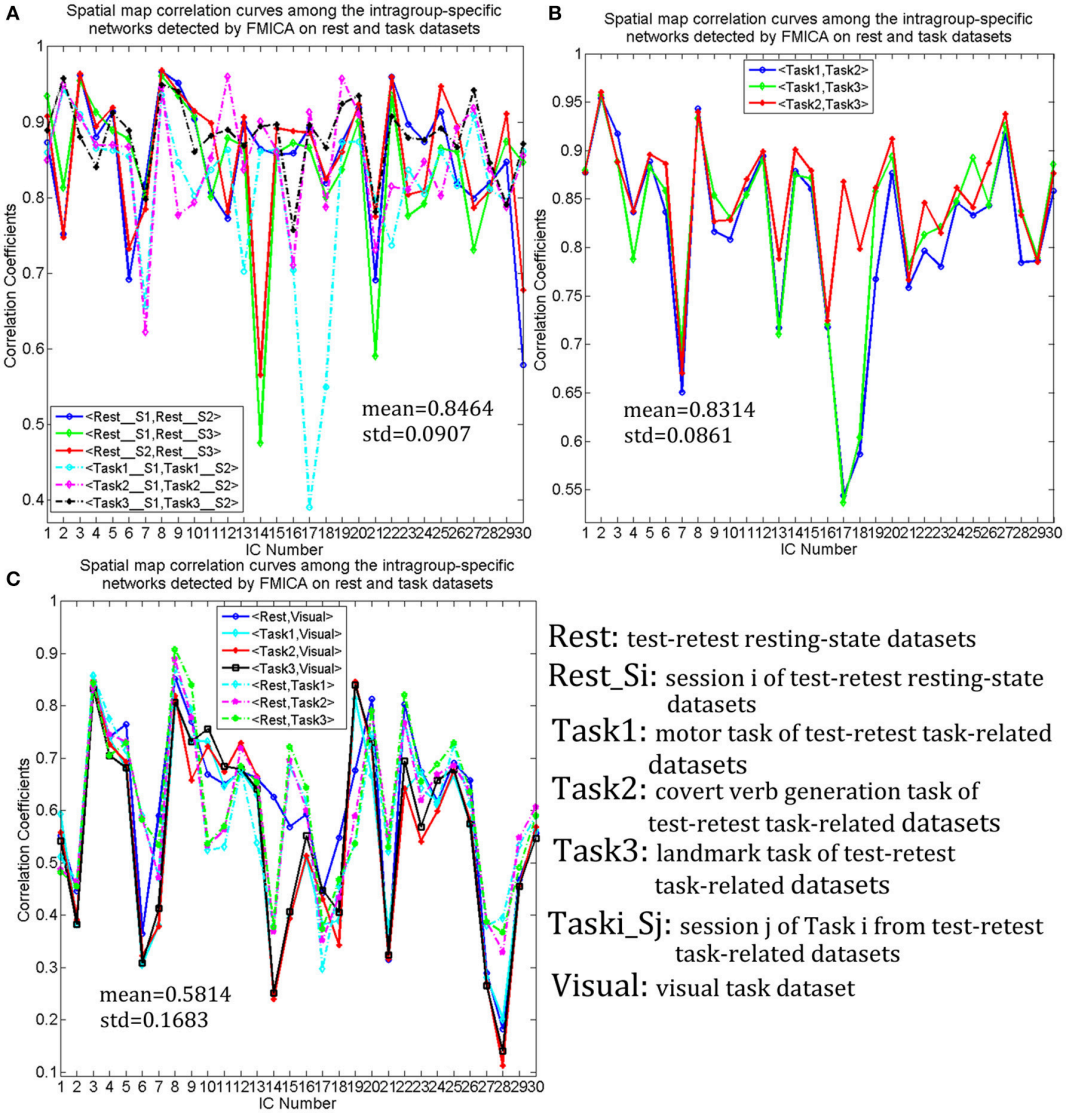
The spatial correlation curves of the intragroup-specific BFNs generated by FMICA among the test-retest resting-state datasets, three test-retest task-related datasets and visual task dataset: **(A)** the spatial map correlation curves among the intragroup-specific BFNs from the same kinds of datasets with different sessions; **(B)** the spatial map correlation curves among the intragroup-specific BFNs with respect to the test-retest task-related datasets; **(C)** the spatial map correlation curves among the intragroup-specific intrinsic BFNs from different kinds of datasets.

Intragroup-specific BFNs for each of the 10 datasets and the corresponding intergroup BFNs, selected visually by the experts from the estimated components by FMICA, were showed in Figure 8 for the first five BFNs due to space limitation, and results for the remaining 25 BFNs were shown in Figure S3. The TD lobes, Brodmann areas, AAL regions and the representative MNI coordinates involved in these BFNs, were recorded in Table S2. It could be observed that most of the intrinsic BFNs extracted in Experiment 2 had high reproducibility in Experiment 3, and better across-sessions than across-datasets consistency of the BFNs was also observed. Correlation analysis was performed to quantify such consistency in various cases. Firstly, mean and std values of correlation coefficients among the intragroup-specific BFNs from different sessions of the resting-state datasets under the same condition (e.g., session 1 and session 2 of resting-state datasets), were calculated and presented in Figure 9A, demonstrating a high mean correlation of 0.8464 and thus implying high across-sessions reproducibility of the BFNs in resting-state datasets (Wang et al., 2016b). Then, the same correlation analysis were performed across the test-retest task datasets of Tasks 1, 2, and 3 from the same subjects, with results presented in Figure 9B, showing also a high mean correlation of 0.8314 and thus implying across-tasks similarity of the intrinsic functional connectivity architecture (Finn et al., 2015). Finally, correlation analysis were performed on completely different kinds of datasets sharing neither sessions nor tasks, with results shown in Figure 9C, indicating a low correlation of 0.5814 inferior to that in Figures 9A,B and thus demonstrating that the proposed FMICA was able to effectively capture the differences of intragroup BFNs across the different kinds of datasets.

To summarize, it could be stated that the proposed FMICA was effective for the intergroup and intragroup-specific analysis, and could characterize the group-specific difference.

## Discussion

In this paper, a BFNs parcellation model based on feature maps, called FMICA, was proposed with demonstrated effectiveness. This FMICA consisted of four main procedures: (1) the first-level ICA decomposition to extract independent component feature maps for each dataset of each subject; (2) the second-level ICA decomposition to obtain the intragroup feature maps for each dataset; (3) the third-level ICA decomposition to acquire intergroup BFNs across multiple datasets; (4) the ICA-R decomposition to extract intragroup-specific BFNs and subject-specific BFNs based on intragroup feature maps and individual IC feature maps, respectively. On one hand, since FMICA used only the feature maps identified by the single-subject level ICA and the subsequent hierarchical processing steps were incorporated for multi-level analysis, it was able to effectively handle the big neuroimaging datasets with different acquisition parameters. On the other hand, the experimental results showed that FMICA had great capability for brain network identification at the subject-specific, intragroup, intragroup-specific and intergroup levels. For example, the results of Figures 3, 6, 7 demonstrated the FMICA's more effective identification ability of the subject-specific BFNs in contrast to the traditional ICA method; based on the results showed in Figure 9, the intragroup-specific BFNs showed the better across-sessions than across-datasets consistency, while the intragroup-specific ones also uncovered group-specific difference in the spatial distribution compared to the intergroup ones (showed in Figure 8 and Figure S3).

### Comparison with other feature-based ICA methods

It was proposed to perform ICA analysis by summarizing fMRI data of each subject as a feature map and applying subsequently traditional ICA algorithms on these feature maps, where features could be the amplitude of low frequency fluctuations (ALFF) maps for resting-state data or T-statistic maps for task-related data, yielding BFNs strikingly similar to but slightly noisier than the results of spatiotemporal group ICA analysis (i.e., TCGICA; Calhoun and Allen, 2013). Very recently, another novel feature-based ICA model using seed-based functional connectivity as summarizing features was proposed (Iraji et al., 2016), with performance highly depending on the choice of seeds. The proposed FMICA in this paper took spatial maps of the independent components of the subjects as feature maps for group analysis. FMICA could produce the comparable intragroup BFNs to the spatiotemporal-domain based group ICA, as shown in Figure S4, implying that it was more effective than the first feature-based ICA model. Meanwhile, FMICA without the seed-based functional connectivity identification procedure was more flexible than the second feature-based ICA model, and it had extra unique advantages of identifying subject-specific, intragroup-specific and intergroup BFNs due to hierarchical processing incorporated in the model.

### Limitations and future research

Single-subject independent components were used as the input feature maps in the proposed FMICA model. However, edges and shapes of the feature-maps could be susceptible to the preprocessing steps in fMRI data analysis, such as the spatial smoothing with FWHM kernels of different size. Therefore, one future research topic on FMICA might be to develop a more robust model to deal with the effects of the preprocessing steps on the feature maps.

Since brain activity at either resting or task states is non-stationary, and it is very important to characterize the dynamics of brain networks (Calhoun et al., 2014). Although static brain functional activity is considered in the current study, the FMICA has also the potential to provide new options to the investigation of the dynamic characteristics of brain networks.

## Conclusion

In this study, we proposed a generalized feature-map based ICA model, named FMICA, which aimed at facing the ever-increasingly big neuroimaging datasets with diverse acquisition parameters. This proposed model was effective to characterize BFNs at the subject-specific, intragroup, intragroup-specific and intergroup levels. The success of FMICA also implied that the feature maps used as the single-subject representatives could not only reduce the high dimensions of the original fMRI data to a small one, but also capture the useful common and distinct properties embedded in each original data. In summary, this proposed FMICA was expected to have wide applications in neuroimaging neuroscience research, e.g., determining individual brain functional ROIs, characterizing differences of BFNs among individual subjects or among the contrast groups, etc.

## Ethics statement

In this study, all the participants included in this study of visual dataset, test-retest task-related datasets and test-retest NYU resting-state datasets provided written informed consent according to procedures approved by the IRB of East China Normal University (ECNU), South East Scotland Research Ethics Committee 01 and New York University (NYU), respectively. Thus, all subjects gave written informed consent in accordance with the Declaration of Helsinki.

## Author contributions

Collection of fMRI data: NW, HY, WZ, and YS. Design of the work: NW and HY. Analysis and interpretation: NW, CC, WZ, YS, and HY. Drafting the article: NW, HY, and CC.

### Conflict of interest statement

The authors declare that the research was conducted in the absence of any commercial or financial relationships that could be construed as a potential conflict of interest.

## References

[B1] Allen E. A. Erhardt E. B. Wei Y. Eichele T. Calhoun V. D. (2012). Capturing inter-subject variability with group independent component analysis of fMRI data: a simulation study. Neuroimage 59, 4141–4159. 10.1016/j.neuroimage.2011.10.010 22019879 PMC3690335

[B2] Bagarinao E. Matsuo K. Nakai T. Sato S. (2003). Estimation of general linear model coefficients for real-time application. Neuroimage 19, 422–429. 10.1016/S1053-8119(03)00081-8 12814591

[B3] Baumgartner R. Ryner L. Richter W. Summers R. Jarmasz M. Somorjai R. (2000). Comparison of two exploratory analysis methods for fMRI: fuzzy clustering vs. principal component analysis. Magn. Reson. Imaging 18, 89–94. 10.1016/S0730-725X(99)00102-2 10642106

[B4] Beckmann C. F. Smith S. M. (2004). Probabilistic independent component analysis for functional magnetic resonance imaging. IEEE Trans. Med. Imaging 23, 137–152. 10.1109/TMI.2003.822821 14964560

[B5] Beckmann C. F. Smith S. M. (2005). Tensorial extensions of independent component analysis for multisubject FMRI analysis. Neuroimage 25, 294–311. 10.1016/j.neuroimage.2004.10.043 15734364

[B6] Beckmann C. F. DeLuca M. Devlin J. T. Smith S. M. (2005). Investigations into resting-state connectivity using independent component analysis. Philos. Trans. R. Soc. Lond. B Biol. Sci. 360, 1001–1013. 10.1098/rstb.2005.1634 16087444 PMC1854918

[B7] Bell A. J. Sejnowski T. J. (1995). An information-maximization approach to blind separation and blind deconvolution. Neural Comput. 7, 1129–1159. 10.1162/neco.1995.7.6.1129 7584893

[B8] Biswal B. B. Ulmer J. L. (1999). Blind source separation of multiple signal sources of fMRI data sets using independent component analysis. J. Comput. Assist. Tomo. 23, 265–271. 10.1097/00004728-199903000-00016 10096335

[B9] Biswal B. Kylen J. V. Hyde J. S. (1997). Simultaneous assessment of flow and BOLD signals in resting-state functional connectivity maps. NMR Biomed. 10, 165–170. 10.1002/(SICI)1099-1492(199706/08)10:4/5<165::AID-NBM454>3.0.CO;2-7 9430343

[B10] Biswal B. Yetkin F. Z. Haughton V. M. Hyde J. S. (1995). Functional connectivity in the motor cortex of resting human brain using echo-planar MRI. Magn. Reson. Med. 34, 537–541. 10.1002/mrm.1910340409 8524021

[B11] Calhoun V. D. Adali T. Pearlson G. D. Pekar J. J. (2001). A method for making group inferences from functional MRI data using independent component analysis. Hum. Brain Mapp. 14, 140–151. 10.1002/hbm.1048 11559959 PMC6871952

[B12] Calhoun V. D. Adali T. (2012). Multisubject independent component analysis of fMRI: a decade of intrinsic networks, default mode, and neurodiagnostic discovery. IEEE Rev. Biomed. Eng. 5, 60–73. 10.1109/RBME.2012.2211076 23231989 PMC4433055

[B13] Calhoun V. D. Allen E. (2013). Extracting intrinsic functional networks with feature-based group independent component analysis. Psychometrika 78, 243–259. 10.1007/s11336-012-9291-3 25107615

[B14] Calhoun V. D. Miller R. Pearlson G. Adali T. (2014). The chronnectome: time-varying connectivity networks as the next frontier in fMRI data discovery. Neuron 84, 262–274. 10.1016/j.neuron.2014.10.015 25374354 PMC4372723

[B15] Calhoun V. D. Potluru V. K. Phlypo R. Silva R. F. Pearlmutter B. A. Caprihan A.. (2013). Independent component analysis for brain fMRI does indeed select for maximal independence. PLoS ONE 8:e73309. 10.1371/annotation/52c7b854-2d52-4b49-9f9f-6560830f9428 24009746 PMC3757003

[B16] Cordes D. Nandy R. R. (2004). Investigating the reliability of ICA sources obtained after PCA preprocessing. Proc. Int. Soc. Magn. Reson. 1086 Available online at: http://cds.ismrm.org/ismrm-2004/Files/001086.pdf

[B17] Cordes D. Haughton V. Carew J. Arfanakis K. Maravilla K. (2002). Hierarchical clustering to measure connectivity in fMRI resting-state data. Magn. Reson. Imaging 20, 305–317. 10.1016/S0730-725X(02)00503-9 12165349

[B18] Damoiseaux J. S. Beckmann C. F. Arigita E. J. Barkhof F. Scheltens P. Stam C. J.. (2008). Reduced resting-state brain activity in the “default network” in normal aging. Cereb. Cortex 18, 1856–1864. 10.1093/cercor/bhm207 18063564

[B19] Damoiseaux J. S. Rombouts S. A. R. B. Barkhof F. Scheltens P. Stam C. J. Smith S. M.. (2006). Consistent resting state networks across healthy subjects. Proc. Natl. Acad. Sci. U.S.A. 103, 13848–13853. 10.1073/pnas.0601417103 16945915 PMC1564249

[B20] De Luca M. Beckmann C. F. De Stefano N. Matthews P. M. Smith S. M. (2006). fMRI resting state networks define distinct modes of long-distance interactions in the human brain. Neuroimage 29, 1359–1367. 10.1016/j.neuroimage.2005.08.035 16260155

[B21] Du Y. Fan Y. (2013). Group information guided ICA for fMRI data analysis. Neuroimage 69, 157–197. 10.1016/j.neuroimage.2012.11.008 23194820

[B22] Erhardt E. B. Allen E. A. Wei Y. Eichele T. Calhoun V. D. (2012). SimTB, a simulation toolbox for fMRI data under a model of spatiotemporal separability. Neuroimage 59, 4160–4167. 10.1016/j.neuroimage.2011.11.088 22178299 PMC3690331

[B23] Esposito F. Scarabino T. Hyvarinen A. Himberg J. Formisano E. Comani S.. (2005). Independent component analysis of fMRI group studies by self-organizing clustering. Neuroimage 25, 193–205. 10.1016/j.neuroimage.2004.10.042 15734355

[B24] Fadili M. J. Ruan S. Bloyet D. Mazoyer B. (2000). A multi-step unsupervised fuzzy clustering analysis of fMRI time series. Hum. Brain Mapp. 10, 160–178. 10.1002/1097-0193(200008)10:4<160::AID-HBM20>3.0.CO;2-U 10949054 PMC6871966

[B25] Finn E. S. Shen X. Scheinost D. Rosenberg M. D. Huang J. Chun M. M.. (2015). Functional connectome fingerprinting: identifying individuals using patterns of brain connectivity. Nat. Neurosci. 18, 1664–1671. 10.1038/nn.4135 26457551 PMC5008686

[B26] Friston K. J. Frith C. D. Turner R. Frackowiak R. S. (1995). Characterizing evoked hemodynamics with fMRI. Neuroimage 2, 157–165. 10.1006/nimg.1995.1018 9343598

[B27] Friston K. J. Holmes A. P. Worsley K. J. Poline J. P. Frith C. D. Frackowiak R. S. (1994). Statistical parametric maps in functional imaging: a general linear approach. Hum. Brain Mapp. 2, 189–210. 10.1002/hbm.460020402

[B28] Georgiev P. Theis F. Cichocki A. Bakardjlan H. (2007). Sparse component analysis: a new tool for data mining, in Data Mining in Biomedicine, Vol. 7, Part 1, eds Pardalos P. M. Boginski V. L. Vazacopoulos A. (New York, NY: Springer), 91–116.

[B29] Golay X. Kollias S. Stoll G. Meier D. Valavanis A. Boesiger P. (1998). A new correlation-based fuzzy logic clustering algorithm for fMRI. Magn. Reson. Med. 40, 249–260. 10.1002/mrm.1910400211 9702707

[B30] Gorgolewski K. J. Storkey A. Bastin M. E. Whittle I. R. Wardlaw J. M. Pernet C. R. (2013). A test-retest fMRI dataset for motor, language and spatial attention functions. Gigascience 2:6. 10.1186/2047-217X-2-6 23628139 PMC3641991

[B31] Greicius M. D. Krasnow B. Reiss A. L. Menon V. (2003). Functional connectivity in the resting brain: a network analysis of the default mode hypothesis. Proc. Natl. Acad. Sci. U.S.A. 100, 253–258. 10.1073/pnas.0135058100 12506194 PMC140943

[B32] Himberg J. Hyvärinen A. Esposito F. (2004). Validating the independent components of neuroimaging time series via clustering and visualization. Neuroimage 22, 1214–1222. 10.1016/j.neuroimage.2004.03.027 15219593

[B33] Hyvärinen A. Oja E. (1997). A fast fixed-point algorithm for independent component analysis. Neural Comput. 9, 1483–1492. 10.1162/neco.1997.9.7.1483 10798706

[B34] Iraji A. Calhoun V. D. Wiseman N. M. Davoodi-Bojd E. Avanaki M. R. Haacke E. M.. (2016). The connectivity domain: analyzing resting state fMRI data using feature-based data-driven and model-based methods. Neuroimage 134, 494–507. 10.1016/j.neuroimage.2016.04.006 27079528 PMC4957565

[B35] Kawashima R. Okuda J. Umetsu A. Sugiura M. Inoue K. Suzuki K.. (2000). Human cerebellum plays an important role in memory-timed finger movement: an fMRI study. J. Neurophysiol. 83, 1079–1087. Available online at: http://jn.physiology.org/content/83/2/1079 10669519 10.1152/jn.2000.83.2.1079

[B36] Kiviniemi V. Biswal B. B. Jauhiainen J. Tervonen O. (2000). Principal component analysis of resting-state fMRI data sets, in Proceedings of the 38th Annual Meeting of ASNR (Atlanta), 295.

[B37] Langers D. R. M. (2010). Unbiased group-level statistical assessment of independent component maps by means of automated retrospective matching. Hum. Brain Mapp. 31, 727–742. 10.1002/hbm.20901 19823986 PMC6870691

[B38] Li Y. O. Adal,ı T. Calhoun V. D. (2007). Estimating the number of independent components for functional magnetic resonance imaging data. Hum. Brain Mapp. 28, 1251–1266. 10.1002/hbm.20359 17274023 PMC6871474

[B39] Lin Q. H. Zheng Y. R. Yin F. L. Liang H. Calhoun V. D. (2007). A fast algorithm for one-unit ICA-R. Inf. Sci. 177, 1265–1275. 10.1016/j.ins.2006.09.011

[B40] Lu W. Rajapakse J. C. (2006). ICA with reference. Neurocomputing 69, 2244–2257. 10.1016/j.neucom.2005.06.021

[B41] Lv J. Jiang X. Li X. Zhu D. Chen H. Zhang T.. (2015a). Sparse representation of whole-brain fMRI signals for identification of functional networks. Med. Image Anal. 20, 112–134. 10.1016/j.media.2014.10.011 25476415

[B42] Lv J. Jiang X. Li X. Zhu D. Zhang S. Zhao S.. (2015b). Holistic atlases of functional networks and interactions reveal reciprocal organizational architecture of cortical function. IEEE Trans. Biomed. Eng. 62, 1120–1131. 10.1109/TBME.2014.2369495 25420254

[B43] Maldjian J. A. Laurienti P. J. Burdette J. H. (2004). Precentral gyrus discrepancy in electronic versions of the talairach atlas. Neuroimage 21, 450–455. 10.1016/j.neuroimage.2003.09.032 14741682

[B44] Maldjian J. A. Laurienti P. J. Burdette J. B. Kraft R. A. (2003). An automated method for neuroanatomic and cytoarchitectonic atlas-based interrogation of fMRI data sets. Neuroimage 19, 1233–1239. 10.1016/S1053-8119(03)00169-1 12880848

[B45] McKeown M. J. Makeig S. Brown G. G. Jung T. P. Kindermann S. S. Bell A. J. (1998). Analysis of fMRI data by blind separation into spatial independent component analysis. Hum. Brain Mapp. 6, 160–188. 10.1002/(SICI)1097-0193(1998)6:3<160::AID-HBM5>3.0.CO;2-1 9673671 PMC6873377

[B46] Menon V. Uddin L. Q. (2010). Saliency, switching, attention and control: a network model of insula function. Brain Struct. Funct. 214, 655–667. 10.1007/s00429-010-0262-0 20512370 PMC2899886

[B47] Minka T. P. (2000). Automatic Choice of Dimensionality for PCA. Technical report 514. MIT.

[B48] Raichle M. E. MacLeod A. M. Snyder A. Z. Powers W. J. Gusnard D. A. Shulman G. L. (2001). A default mode of brain function. Proc. Natl. Acad. Sci. U.S.A. 98, 676–682. 10.1073/pnas.98.2.676 11209064 PMC14647

[B49] Ren T. Zeng W. Wang N. Chen L. Wang C. (2014). A novel approach for fMRI data analysis based on the combination of sparse approximation and affinity propagation clustering. Magn. Reson. Imaging 32, 736–746. 10.1016/j.mri.2014.02.023 24721006

[B50] Schmithorst V. J. Holland S. K. (2004). Comparison of three methods for generating group statistical inferences from independent component analysis of functional magnetic resonance imaging data. J. Magn. Reson. Imaging 19, 365–368. 10.1002/jmri.20009 14994306 PMC2265794

[B51] Schöpf V. Kasess C. H. Lanzenberger R. Fischmeister F. Windischberger C. Moser E. (2010). Fully exploratory network ICA (FENICA) on resting-state fMRI data. J. Neurosci. Methods 192, 207–213. 10.1016/j.jneumeth.2010.07.028 20688104

[B52] Shehzad Z. Kelly A. C. Reiss P. T. Gee D. G. Gotimer K. Uddin L. Q.. (2009). The resting brain: unconstrained yet reliable. Cereb. Cortex 19, 2209–2229. 10.1093/cercor/bhn256 19221144 PMC3896030

[B53] Shi Y. Zeng W. Wang N. Chen D. (2015a). A novel fMRI group data analysis method based on data-driven reference extracting from group subjects. Comput. Methods Programs Biomed. 122, 362–371. 10.1016/j.cmpb.2015.09.002 26387634

[B54] Shi Y. Zeng W. Wang N. Wang S. Huang Z. (2015b). Early warning for human mental sub-health based on fMRI data analysis: an example from a seafarers' resting-data study. Front. Psychol. 6:1030. 10.3389/fpsyg.2015.01030 26257686 PMC4511829

[B55] Svensén M. Kruggel F. Benali H. (2002). ICA of fMRI group study data. Neuroimage 16, 551–563. 10.1006/nimg.2002.1122 12169242

[B56] Tang X. Zeng W. Wang N. Yang J. (2015). An adaptive RV measure based fuzzy weighting subspace clustering (ARV-FWSC) for fMRI data analysis. Biomed. Signal Process. Control 22, 146–154. 10.1016/j.bspc.2015.07.006

[B57] Uddin L. Q. (2015). Salience processing and insular cortical function and dysfunction. Nat. Rev. Neurosci. 16, 55–61. 10.1038/nrn3857 25406711

[B58] Valente G. Martino F. D. Filosa G. Balsi M. Formisano E. (2009). Optimizing ICA in fMRI using information on spatial regularities of the sources. Magn. Reson. Imaging 27, 1110–1119. 10.1016/j.mri.2009.05.036 19570634

[B59] Wang N. Zeng W. Chen D. (2016a). A Novel Sparse Dictionary Learning Separation (SDLS) Model with Adaptive Dictionary Mutual Incoherence Constraint for fMRI Data Analysis. IEEE Trans. Biomed. Eng. 63, 2376–2389. 10.1109/TBME.2016.2533722 26929024

[B60] Wang N. Zeng W. Chen L. (2012). A Fast-FENICA method on resting state fMRI data. J. Neurosci. Methods 209, 1–12. 10.1016/j.jneumeth.2012.05.007 22659001

[B61] Wang N. Zeng W. Chen L. (2013). SACICA: A sparse approximation coefficients based ICA model for functional magnetic resonance imaging data analysis. J. Neurosci. Methods 216, 49–61. 10.1016/j.jneumeth.2013.03.014 23563324

[B62] Wang N. Zeng W. Chen D. Yin J. Chen L. (2016b). A novel brain networks enhancement model (BNEM) for BOLD fMRI data analysis with highly spatial reproducibility. IEEE J. Biomed. Health Inform. 20, 1107–1119. 10.1109/JBHI.2015.2439685 26054077

[B63] Wang N. Zeng W. Shi Y. Ren T. Jing Y. Yin J.. (2015). WASICA: An effective wavelet-shrinkage based ICA model for brain fMRI data analysis. J. Neurosci. Methods 246, 75–96. 10.1016/j.jneumeth.2015.03.011 25791013

[B64] Wang Z. Xia M. Jin Z. Yao L. Long Z. (2014). Temporally and spatially constrained ICA of fMRI data analysis. PLoS ONE, 9:e94211. 10.1371/journal.pone.0094211 24727944 PMC3984144

[B65] Yan C. G. Zang Y. F. (2010). DPARSF: a MATLAB toolbox for “pipeline” data analysis of resting-state fMRI. Front. Syst. Neurosci. 4:13 10.3389/fnsys.2010.00013 20577591 PMC2889691

[B66] Yang Z. Chang C. Xu T. Jiang L. Handwerker D. A. Castellanos F. X.. (2014). Connectivity trajectory across lifespan differentiates the precuneus from the default network. Neuroimage 89, 45–56. 10.1016/j.neuroimage.2013.10.039 24287438 PMC3944140

[B67] Yeo B. T. Krienen F. M. Sepulcre J. Sabuncu M. R. Lashkari D. Hollinshead M.. (2011). The organization of the human cerebral cortex estimated by intrinsic functional connectivity. J. Neurophysiol. 106, 1125–1165. 10.1152/jn.00338.2011 21653723 PMC3174820

[B68] Zhang J. Li D. Chen H. Fang F. (2011). Analysis of activity in fMRI data using affinity propagation clustering. Comput. Methods Biomech. Biomed. Eng. 14, 271–281. 10.1080/10255841003766829 21347914

[B69] Zuo X. N. Kelly C. Adelstein J. S. Klein D. F. Castellanos F. X. Milham M. P. (2010). Reliable intrinsic connectivity networks: test-retest evaluation using ICA and dual regression approach. Neuroimage 49, 2163–2177. 10.1016/j.neuroimage.2009.10.080 19896537 PMC2877508

