# Supplementary material for: A Novel Feature-Map Based ICA Model for Identifying the Individual, Intra/Inter-Group Brain Networks across Multiple fMRI Datasets
Source: Front Neurosci. 2017 Sep 8;11:510. doi: 10.3389/fnins.2017.00510 (PMC5596109; doi:10.3389/fnins.2017.00510)
Supplement: Supplementary file 1 [file Presentation1.PDF]

Supplementary files for FMICA

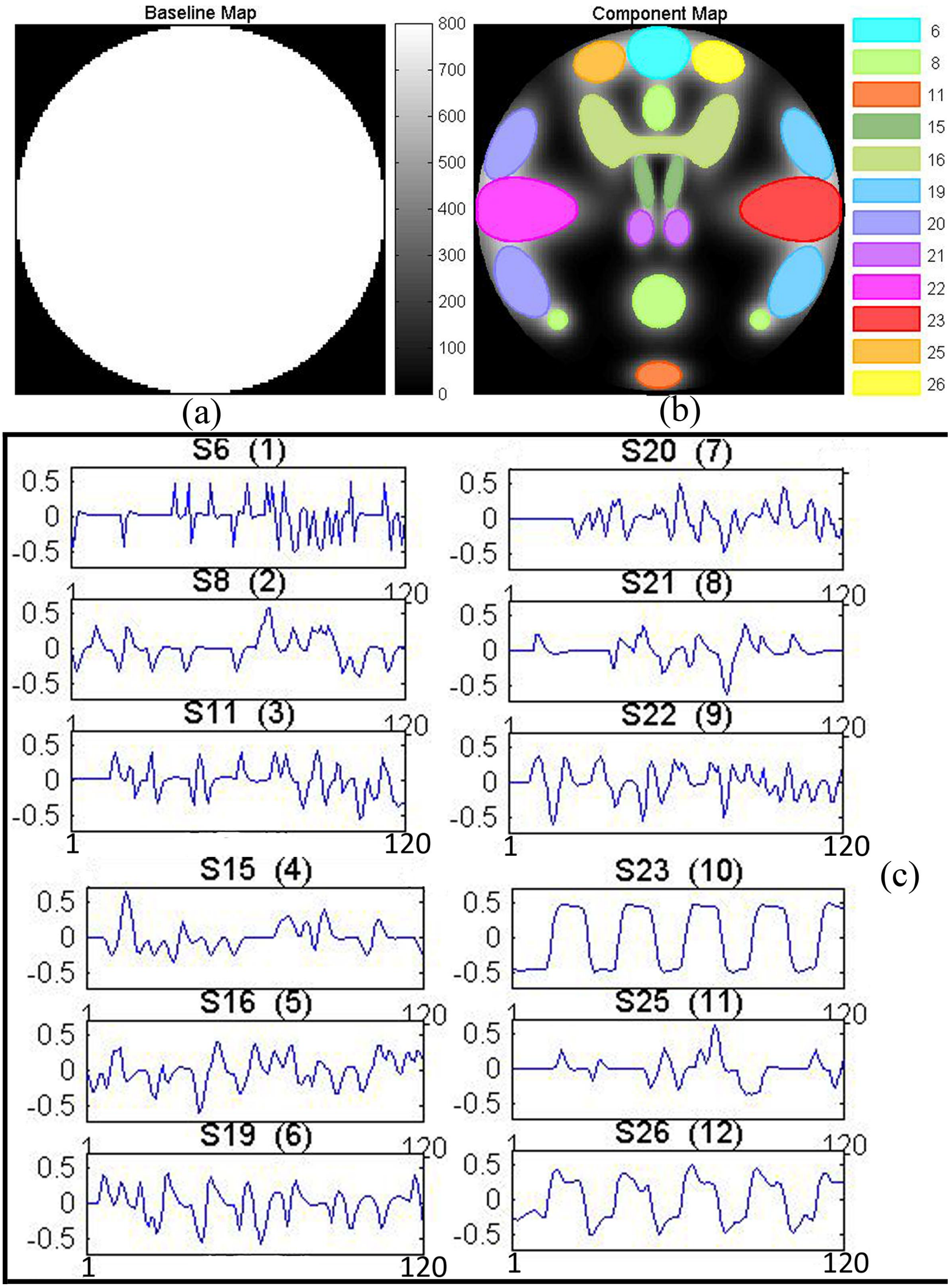

Fig. S1 Simulation dataset: (a) baseline map; (b) the spatial distribution of the 12 sources selected from the default source template provided by SimTB, marked in 12

distinct colors; (c) the corresponding TCs of the 12 sources; (d) the random spatial variant behaviors of the simulated sources in translation, rotation, expansion or contraction views for each subject.

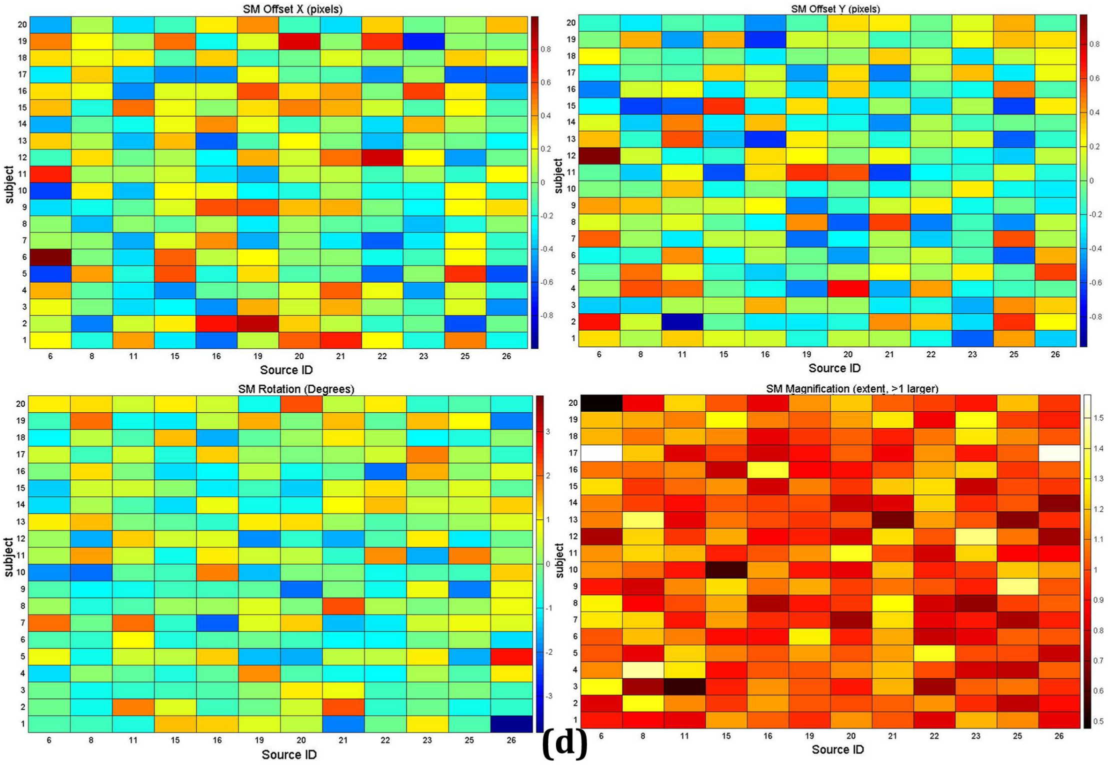

Fig. S1 (cont.)

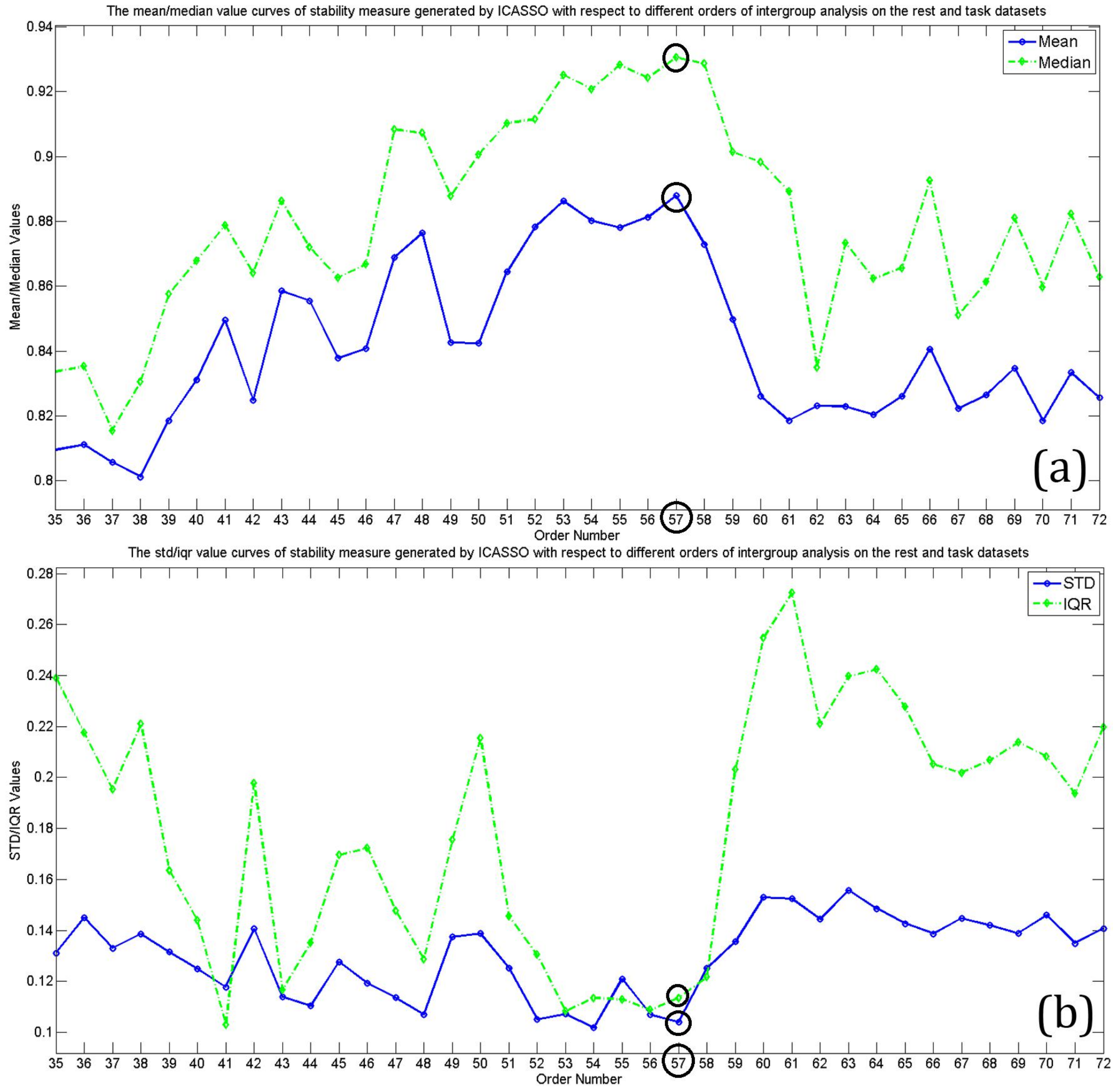

Fig. S2 Statistical curves of the stability measure generated by ICASSO on the aggregated intragroup-level feature maps from the test-rest resting-state datasets, test-retest task-related datasets and the visual task dataset in Experiment 3. ICASSO ran with the order number changing from the minimum number (35) to the maximum one (72).

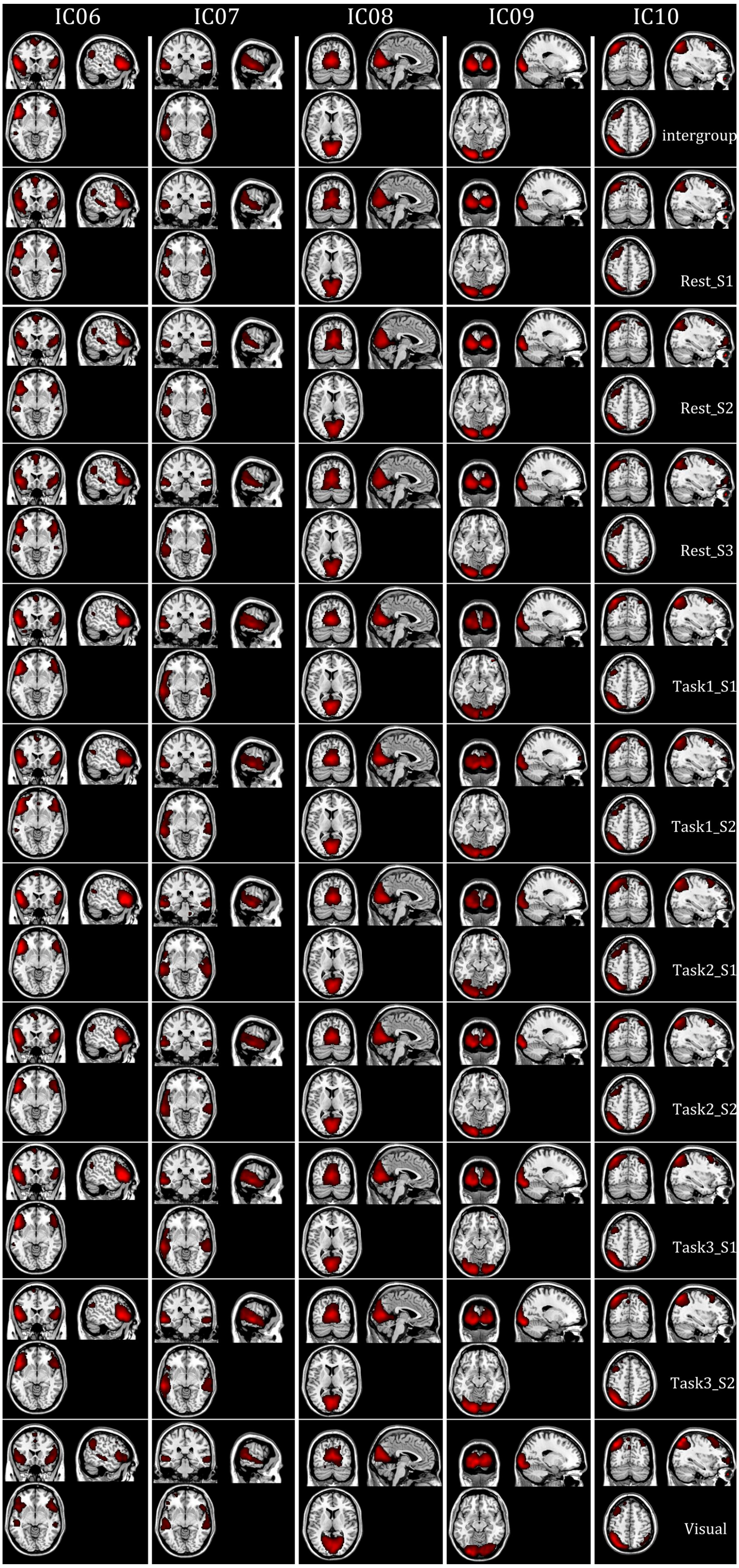
IC06
IC07
IC08
IC09
IC10
intergroup
Rest_S1
Rest_S2
Rest_S3
Task1_S1
Task1_S2
Task2_S1
Task2_S2
Task3_S1
Task3_S2
Visual

Fig. S3 The spatial map distribution of other twenty-five intrinsic BFNs at the intergroup and intragroup-specific levels in Experiment 3: each column depicted a BFN at the intergroup and intragroup-specific levels; Rest_S i denoted the *ith* session of test-retest resting-state datasets; Task i_S j denoted the *jth* session of Task i from the test-retest task-related datasets; Visual denoted the visual task dataset.

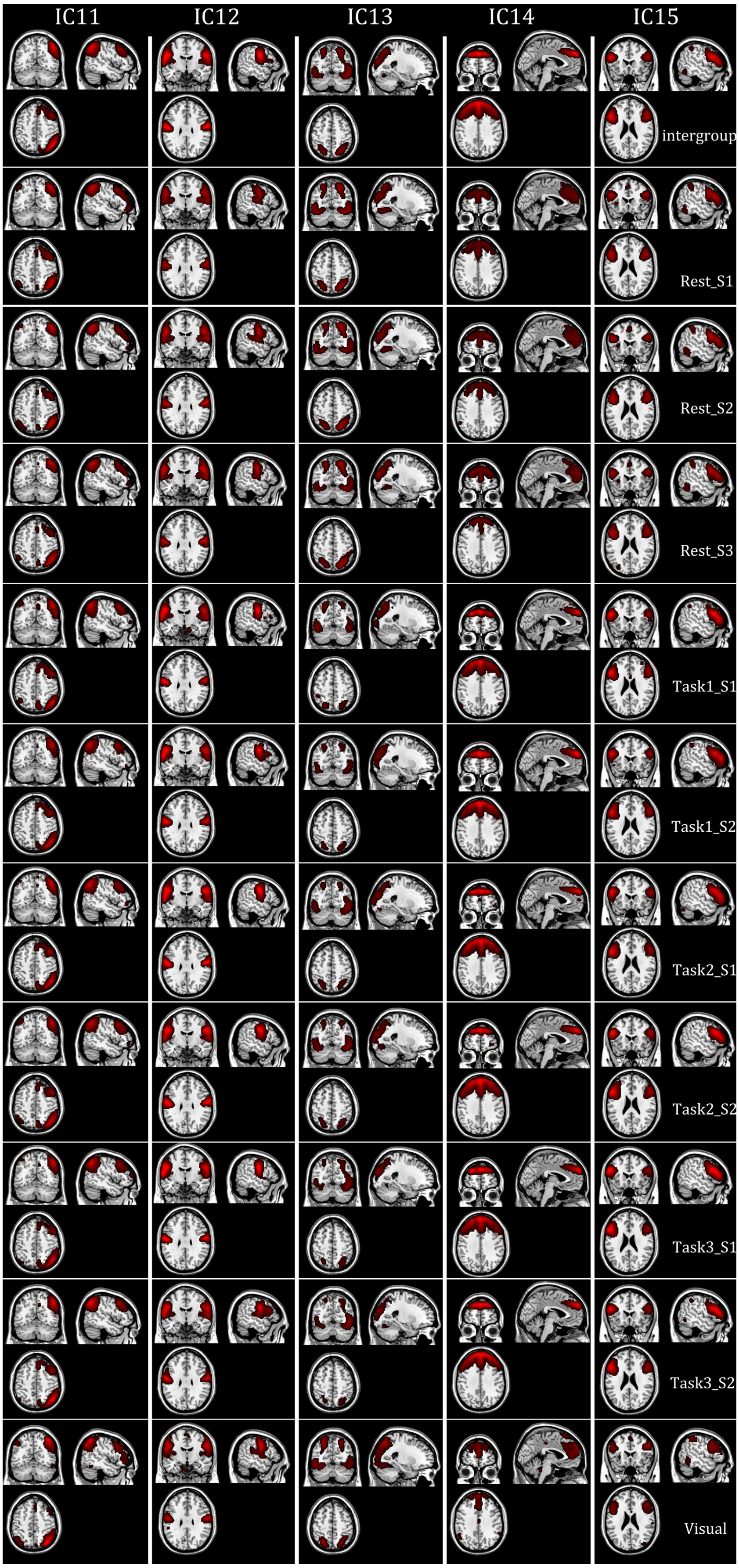
IC11
IC12
IC13
IC14
IC15
intergroup
Rest_S1
Rest_S2
Rest_S3
Task1_S1
Task1_S2
Task2_S1
Task2_S2
Task3_S1
Task3_S2
Visual

Fig. S3 (cont.)

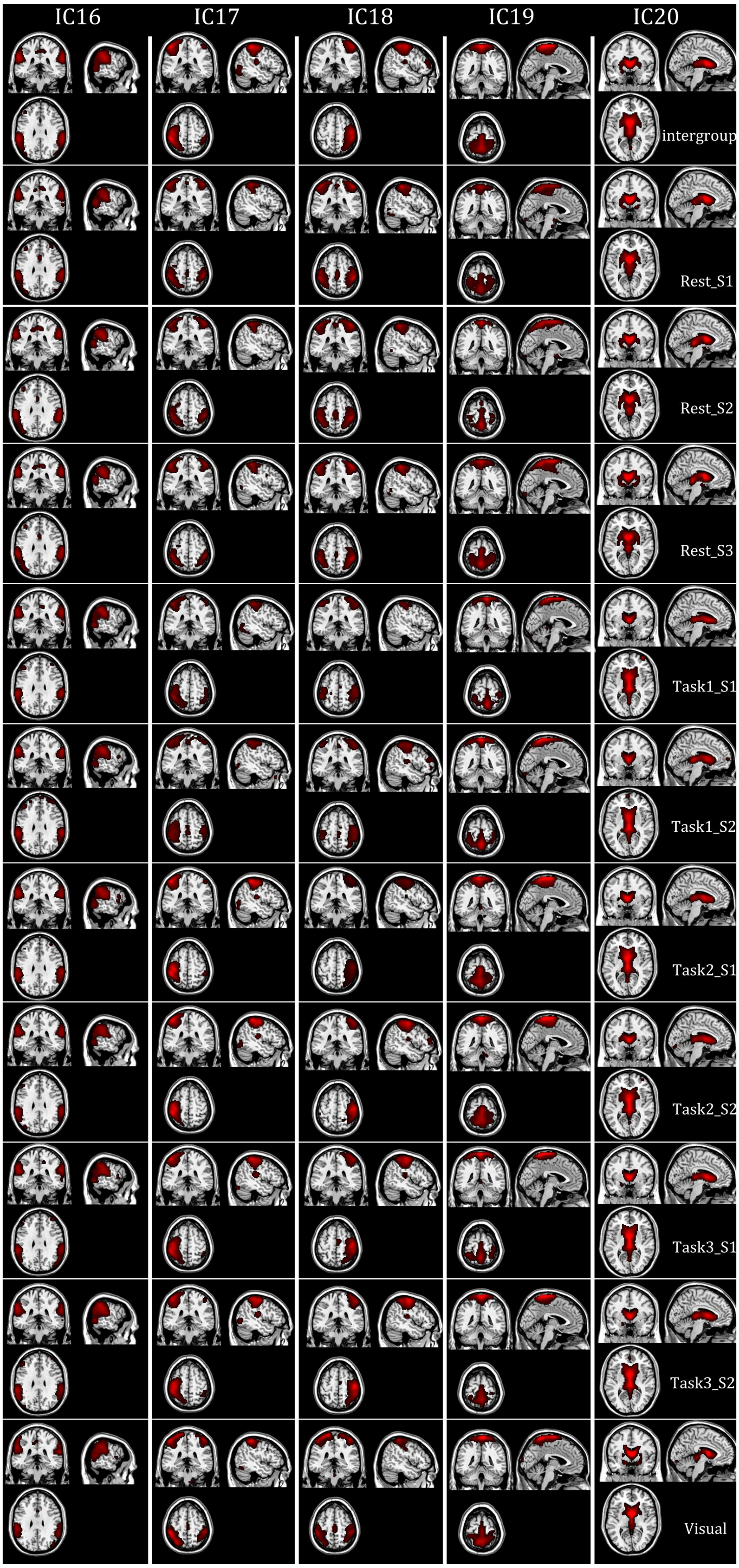
IC16
IC17
IC18
IC19
IC20
intergroup
Rest_S1
Rest_S2
Rest_S3
Task1_S1
Task1_S2
Task2_S1
Task2_S2
Task3_S1
Task3_S2
Visual

Fig. S3 (cont.)

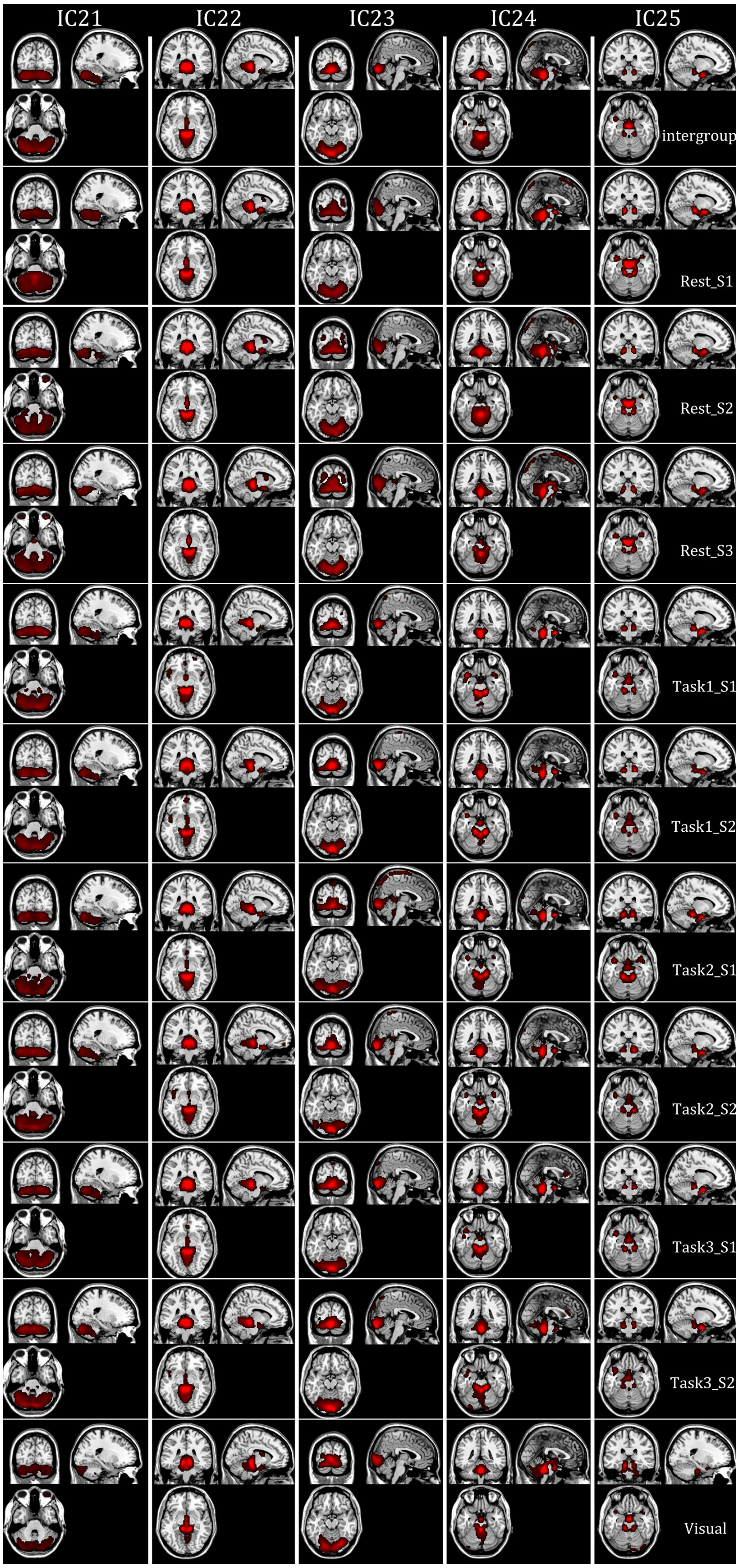
IC21
IC22
IC23
IC24
IC25
intergroup
Rest_S1
Rest_S2
Rest_S3
Task1_S1
Task1_S2
Task2_S1
Task2_S2
Task3_S1
Task3_S2
Visual

Fig. S3 (cont.)

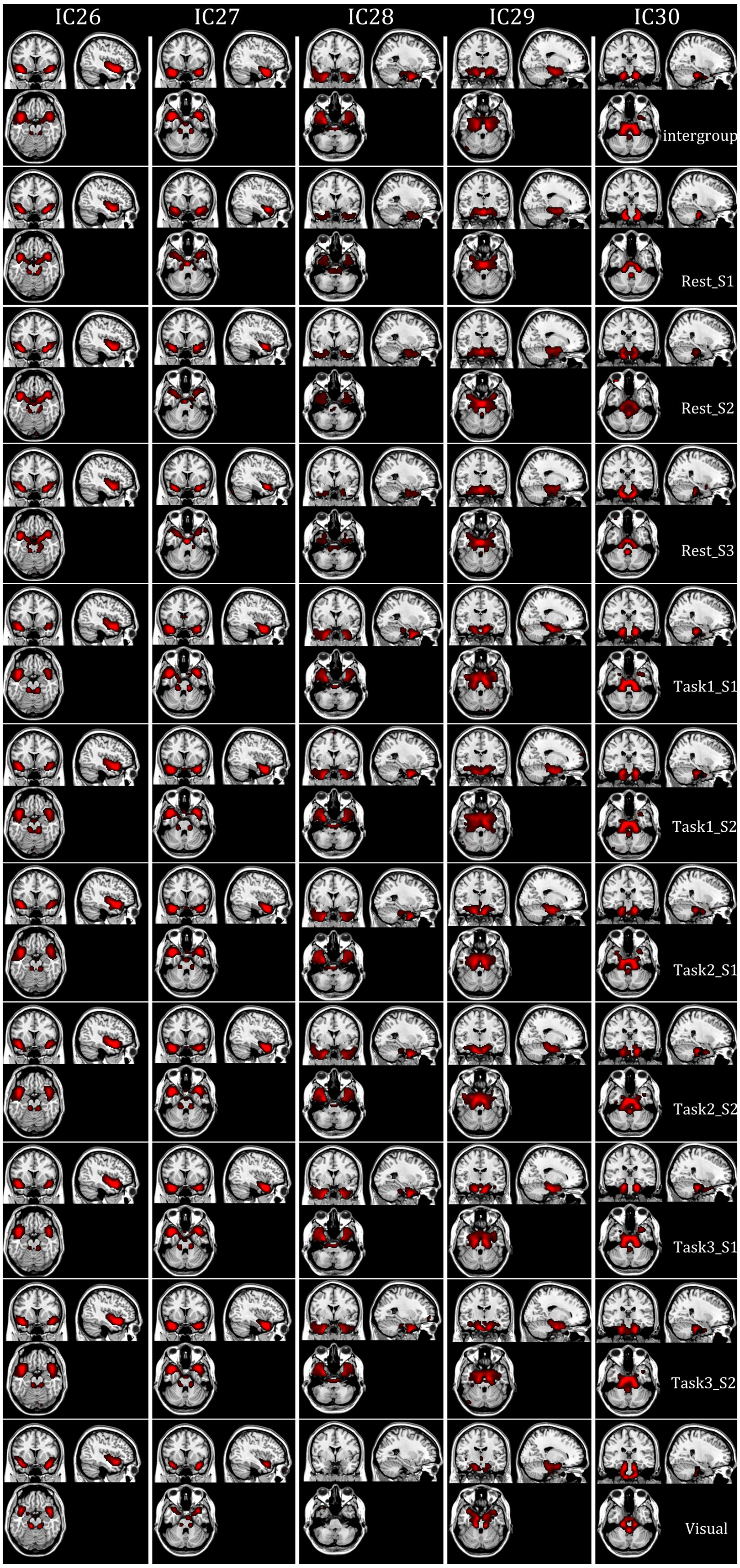
IC26
IC27
IC28
IC29
IC30
intergroup
Rest_S1
Rest_S2
Rest_S3
Task1_S1
Task1_S2
Task2_S1
Task2_S2
Task3_S1
Task3_S2
Visual

Fig. S3 (cont.)

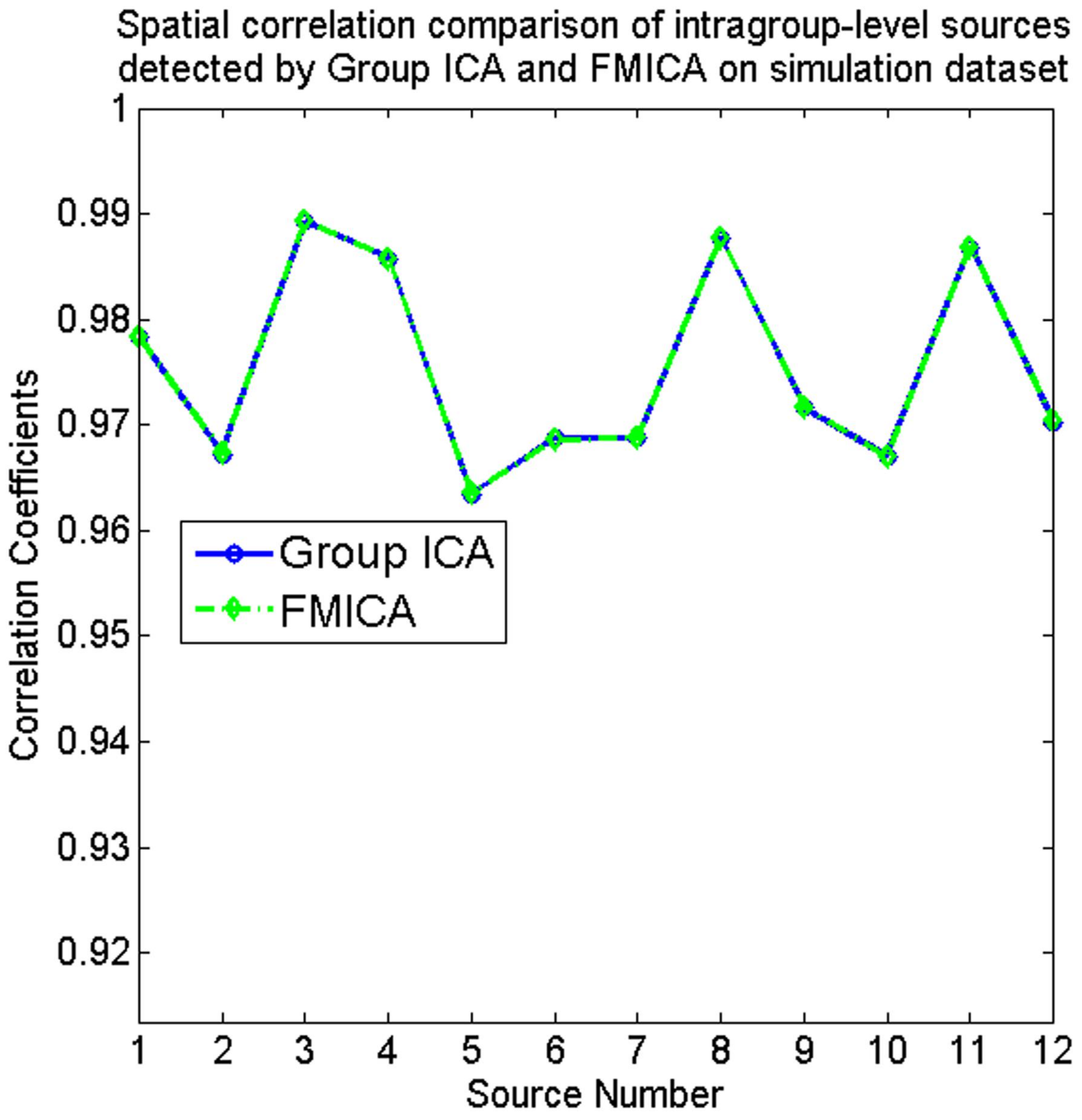

Fig. S4 The comparative curves of spatial correlation regarding the intragroup sources detected by Group ICA on the simulation dataset and FMICA on feature maps generated from the simulation dataset, respectively.

Table S1. The correlation analysis results of the estimated intragroup-level BFNs at the different ratios of the retained independent components of the intragroup level and to that of the individual level on simulation dataset with twenty subjects. For simulation dataset, the true source number for each subject was thirteen, consisting of twelve designed sources and one background source. The mean correlation value at the ratio equal to 1/20 was significantly larger than the ones when the ratio was more than 4/20, as revealed by the two sample T-Tests with significance level equal to 0.05.

**Ratios**	**Component number of intragroup level**	**Components number of individual subject**	**Correlation** (Mean±std)
1/80	13	52	0.9754±0.0095
1/60	13	39	0.9754±0.0095
1/40	13	26	0.9754±0.0095
**1/20**	**13**	**13**	**0.9754±0.0095**
2/20	26	13	0.9751±0.0096
3/20	39	13	0.9748±0.0096
4/20	52	13	0.9704±0.0082
5/20	65	13	0.9505±0.0251
6/20	78	13	0.9271±0.0453
7/20	91	13	0.8973±0.0583

Table S2. The location information of the thirty intrinsic BFNs from the test-retest resting-state datasets, the test-retest task-related datasets for motor, language and spatial attention and the visual task dataset shown in Fig.8 and Fig.S3: the MNI coordinates (in mm) of each network, involved brain lobes, Brodmann areas and AAL atlas regions for each network.

**IC number**	**Representative MNI coordinates (X,Y,Z) in mm**	**TD lobes / Brodmann areas /AAL atlas**
IC01	3, 57, 3	Frontal Lobe /Brodmann Area 9, 10, 24, 32 /Medial Frontal Gyrus, Anterior Cingulate, Superior Frontal Gyrus
IC02	-3, 39, -15	Frontal Lobe /Brodmann Area 10, 11, 24, 25, 32/ Orbital Gyrus, Medial Frontal Gyrus, Anterior Cingulate
IC03	0, -72, 36	Parietal Lobe, Limbic Lobe/Brodmann Area 7, 19, 23, 24, 31/Precuneus, Posterior Cingulate, Cingulate Gyrus
IC04	3, -57, 15	Parietal lobe, Frontal lobe, Temporal lobe, Occipital lobe, Limbic Lobe /Brodmann Area 7, 19, 23, 29, 30, 31, 39 /Posterior Cingulate, Precuneus, Angular Gyrus, Parahippocampal Gyrus, Cingulate Gyrus, Superior Occipital Gyrus, Middle Temporal Gyrus
IC05	-60, -18, 9	Temporal lobe, Insula/Brodmann Area 13, 21, 22, 38, 40, 41, 42 /Superior Temporal Gyrus, Transverse Temporal Gyrus, Middle Temporal Gyrus
IC06	-51, 21, -6	Temporal lobe, Frontal lobe /Brodmann Area 10, 22, 38, 44, 45, 46, 47 /Superior Temporal Gyrus, Inferior Frontal Gyrus, Middle Frontal Gyrus
IC07	-63, -30, -6	Temporal lobe /Brodmann Area 21, 22, 38, 39, 40, 42/ Middle Temporal Gyrus, Superior Temporal Gyrus, Supramarginal Gyrus
IC08	3, -75, 9	Parietal Lobe, Limbic Lobe /Brodmann Area 7, 17, 18, 19, 23, 30/ Posterior Cingulate, Lingual Gyrus, Precuneus, Parahippocampal Gyrus
IC09	-18, -96, -12	Occipital Lobe /Brodmann Area 17, 18, 19 /Inferior Occipital Gyrus, Lingual Gyrus, Middle Occipital Gyrus
IC10	-33, -72, 51	Parietal Lobe /Brodmann Area 7, 19, 39, 40/ Superior Parietal Lobule, Inferior Parietal Lobule, Precuneus, Medial Frontal Gyrus
IC11	48, -60, 48	Parietal Lobe /Brodmann Area 7, 19, 39, 40/ Superior Parietal Lobule, Inferior Parietal Lobule, Supramarginal Gyrus, Angular Gyrus, Precuneus
IC12	-54, -9, 30	Frontal Lobe, Parietal Lobe, Temporal Lobe/Brodmann Area 1, 3, 4, 6, 9, 13,22, 42, 43, 44/ Precentral Gyrus, Postcentral Gyrus, Transverse Temporal Gyrus, Superior Temporal Gyrus, Inferior Frontal Gyrus
IC13	-24, -66, 54	Parietal Lobe, Temporal Lobe, Occipital Lobe /Brodmann Area 7, 18, 19, 37 /Fusiform Gyrus, Precuneus, Inferior Temporal Gyrus, Middle Occipital Gyrus
IC14	-3, 57, 36	Frontal Lobe /Brodmann Area 6, 8, 9, 10, 46/ Superior Frontal Gyrus, Medial Frontal Gyrus, Middle Frontal Gyrus, Precentral Gyrus

IC15	-51, 21, 24	Frontal Lobe /Brodmann Area 6, 9, 10, 44, 45, 46 /Inferior Frontal Gyrus, Middle Frontal Gyrus, Sub-Gyral
IC16	-60, -36, 30	Parietal Lobe, Temporal Lobe, Insula /Brodmann Area 1, 2, 13, 22, 39, 40,42, 43 /Inferior Parietal Lobule, Postcentral Gyrus, Supramarginal Gyrus, Superior Temporal Gyrus
IC17	-48, -36, 60	Parietal Lobe /Brodmann Area 1, 2, 3, 4, 5, 6, 7, 40 /Inferior Parietal Lobule, Postcentral Gyrus, Precentral Gyrus, Superior Parietal Lobule
IC18	48, -36, 60	Parietal Lobe /Brodmann Area 1, 2, 3, 4, 5, 6, 40 /Precentral Gyrus, Postcentral Gyrus, Inferior Parietal Lobule
IC19	-3, -42, 69	Frontal Lobe /Brodmann Area 3, 4, 5, 6, 7 /Medial Frontal Gyrus, Paracentral Lobule, Postcentral Gyrus
IC20	-6, 0, 6	Sub-lobar /Brodmann Area 25 /Caudate, Thalamus, Anterior Cingulate,
IC21	-21, -78, -33	Inferior Semi-Lunar Lobule, Cerebellar Tonsil, Culmen, Vermis
IC22	-9, -36, -9	Limbic Lobe /Brodmann Area 27, 30, 35 /Culmen, Parahippocampal Gyrus, Cerebellar Lingual, Thalamus
IC23	3, -87, -15	Occipital Lobe /Brodmann Area 17, 18, 19 /Lingual Gyrus, Fusiform Gyrus, Cuneus, Culmen, Sub-Gyral, Middle Occipital Gyrus, Vermis
IC24	0, -45, -24	Cerebellar Lingual, Culmen, Vermis
IC25	-12, -30, -21	Limbic Lobe /Culmen, Parahippocampal Gyrus, Superior Temporal Gyrus
IC26	-42, 12, -18	Frontal Lobe, Temporal Lobe, Insula/ Brodmann Area 13, 21, 22, 38, 47/ Superior Temporal Gyrus, Sub-Gyral, Inferior Frontal Gyrus, Middle Temporal Gyru, Culmen, Parahippocampal Gyrus
IC27	-39, 12, -30	Temporal Lobe, Limbic Lobe /Brodmann Area 21, 28, 34, 38 /Superior Temporal Gyrus, Middle Temporal Gyrus, Sub-Gyral, Uncus, Culmen
IC28	-27, 0, -42	Temporal Lobe, Limbic Lobe/Brodmann Area 20, 21, 28, 36, 38 /Uncus, Sub-Gyral, Inferior Temporal Gyrus, Middle Temporal Gyrus, Superior Temporal Gyrus, Parahippocampal Gyrus
IC29	-18, -15, -27	Temporal Lobe, Limbic Lobe/ Brodmann Area 28, 34, 35, 36/ Parahippocampal Gyrus, Uncus, Culmen, Superior Temporal Gyrus, Sub-Gyral
IC30	24, -27, -33	Temporal Lobe, Limbic Lobe /Brodmann Area 28, 35, 36, 38/ Culmen, Parahippocampal Gyrus, Uncus, Superior Temporal Gyrus
